# USP5 inhibition via bone marrow-targeted engineered exosomes for myeloproliferative neoplasms therapy

**DOI:** 10.1186/s12951-025-03588-4

**Published:** 2025-07-11

**Authors:** Wenjun Wang, Yufeng Jiang, Donglei Zhang, Xian Zhang, Qian Liang, Jun Shi, Yuan Zhou, Fuling Zhou

**Affiliations:** 1 Department of Hematology, Zhongnan Hospital of Wuhan University, Wuhan University, Wuhan, 430071 China; 2 State Key Laboratory of Experimental Hematology, Haihe Laboratory of Cell Ecosystem, Institute of Hematology & Blood Diseases Hospital, National Clinical Research Center for Blood Diseases, Chinese Academy of Medical Sciences & Peking Union Medical College, Tianjin, 300020 China; 3 Tianjin Institutes of Health Science, Tianjin, 301600 China; 4 School of Information and Mathematics, Yangtze University, Jingzhou, Hubei 434000 China; 5 Zhoukou Central Hospital, Zhoukou, China

**Keywords:** Myeloproliferative neoplasms, Mesenchymal stem cells, Deubiquitinating enzyme, Bone marrow targeting

## Abstract

Myeloproliferative neoplasms (MPNs) are challenging to treat due to the complex bone marrow (BM) microenvironment and lack of curative therapies. Current treatments fail to eliminate malignant clones and face issues like drug resistance. This study addressed these challenges by identifying USP5 as a critical regulator in *JAK2*^V617F^-mutated mesenchymal stem cells (MSCs), which promotes proliferation by suppressing Caspase-3-mediated apoptosis. We developed engineered exosomes (USP5@Exosome-CP) co-expressing CXCR4 and a P-selectin-targeting peptide to enhance BM targeting. These exosomes, loaded with the USP5 inhibitor USP5-IN-1, demonstrated efficient BM homing and sustained drug release. In MPN mouse models, USP5@Exosome-CP significantly reduced MSC proliferation, extended survival, and showed minimal systemic toxicity. Transcriptomic analysis revealed that USP5 knockdown activated apoptosis pathways and suppressed oncogenic signaling. Our results establish USP5 as a therapeutic target and validate the engineered exosome platform as a promising strategy for MPN treatment, offering a blueprint for targeting other hematologic malignancies. This approach combines USP5 inhibition with BM-targeted nanotechnology, providing a proof-of-concept for personalized MPN therapy with improved efficacy and reduced off-target effects.

**Supplementary Information:**

The online version contains supplementary material available at 10.1186/s12951-025-03588-4.

## Introduction

Myeloproliferative neoplasms (MPN) are a group of clonal hematopoietic stem cell disorders characterized by the overproduction of mature myeloid blood cells, including red blood cells, white blood cells, and platelets [1, 2]. These disorders, which include polycythemia vera (PV), essential thrombocythemia (ET), and primary myelofibrosis (PMF), are driven by acquired somatic mutations in genes such as *JAK2*, *CALR*, or *MPL*, leading to hyperactivation of the JAK/STAT signaling pathway [3, 4]. The *JAK2*^V617F^ mutation, in particular, is a prevalent driver mutation in Philadelphia chromosome-negative MPN (Ph^−^ MPN) and significantly accelerates disease progression by promoting clonal expansion of hematopoietic stem cells [5, 6].

Current treatment options for MPN are primarily palliative, aimed at managing symptoms and reducing thrombotic risks rather than curing the disease [7]. Hydroxyurea, an oral chemotherapy agent, is widely used to reduce blood cell counts and alleviate symptoms [8]. Ruxolitinib, a JAK inhibitor, suppresses hyperactive JAK/STAT signaling and is particularly beneficial for patients with PV and ET [9, 10]. However, these therapies fail to eliminate malignant clones, often lead to dose-limiting toxicities, and face challenges such as drug resistance and disease relapse [11]. Moreover, the chronic nature of MPN and the complexity of its bone marrow (BM) microenvironment further complicate therapeutic interventions.

The BM microenvironment plays a pivotal role in MPN pathogenesis, providing a niche that supports the survival and proliferation of mutant hematopoietic stem cells [12]. Targeted drug delivery to this site remains a major hurdle due to physiological barriers and poor drug penetration [13–15]. The CXCR4-CXCL12 (SDF-1) axis, a key mediator of cell homing and retention in the BM, is critical for cancer cell proliferation, immune evasion, and stromal interactions [16]. CXCR4 is highly expressed on malignant cells in MPN, while CXCL12 is abundantly secreted by BM stromal cells, making this axis a promising target for disrupting pathological cell-matrix interactions [17].

To address these challenges, stem cell-derived exosomes have emerged as innovative drug delivery vehicles [18]. Exosomes are natural nanoscale extracellular vesicles capable of transporting proteins, lipids, and nucleic acids between cells, offering advantages such as biocompatibility, low immunogenicity, and the ability to cross biological barriers [19, 20]. Engineered exosomes can inherit targeting motifs from parental cells, enabling precise delivery to specific tissues while minimizing off-target effects [21, 22]. Recent studies highlight their potential in modulating inflammatory responses, promoting tissue repair, and enhancing drug bioavailability in hard-to-reach niches like the BM [23, 24].

To systematically identify JAK2^V617F^-specific vulnerabilities within the ubiquitin-proteasome system, we employed comprehensive functional screening of 86 deubiquitinating enzymes (DUBs) in mutant versus normal mesenchymal stem cells (MSCs). This unbiased approach was critical because: (i) canonical DUBs (e.g., USP9X/USP15) show disease-context dysregulation not extrapolatable to JAK2^V617F^ MPN; (ii) the mutation remodels ubiquitin signaling in a target-specific manner, necessitating empirical discovery; and (iii) it circumvents presupposition bias to reveal novel targets with mechanistic and therapeutic uniqueness. Through transcriptomic and proteomic analyses, we discovered that USP5 is markedly overexpressed in mutant MSCs (Mut-MSCs), where it suppresses Caspase-3-mediated apoptosis and drives pathological proliferation. Knockdown of USP5 or pharmacological inhibition using USP5-IN-1 restored apoptosis, normalized cell cycle progression, and significantly attenuated Mut-MSC proliferation in vitro and in vivo. These findings establish USP5 as a critical therapeutic target in MPN.

**Scheme 1:**
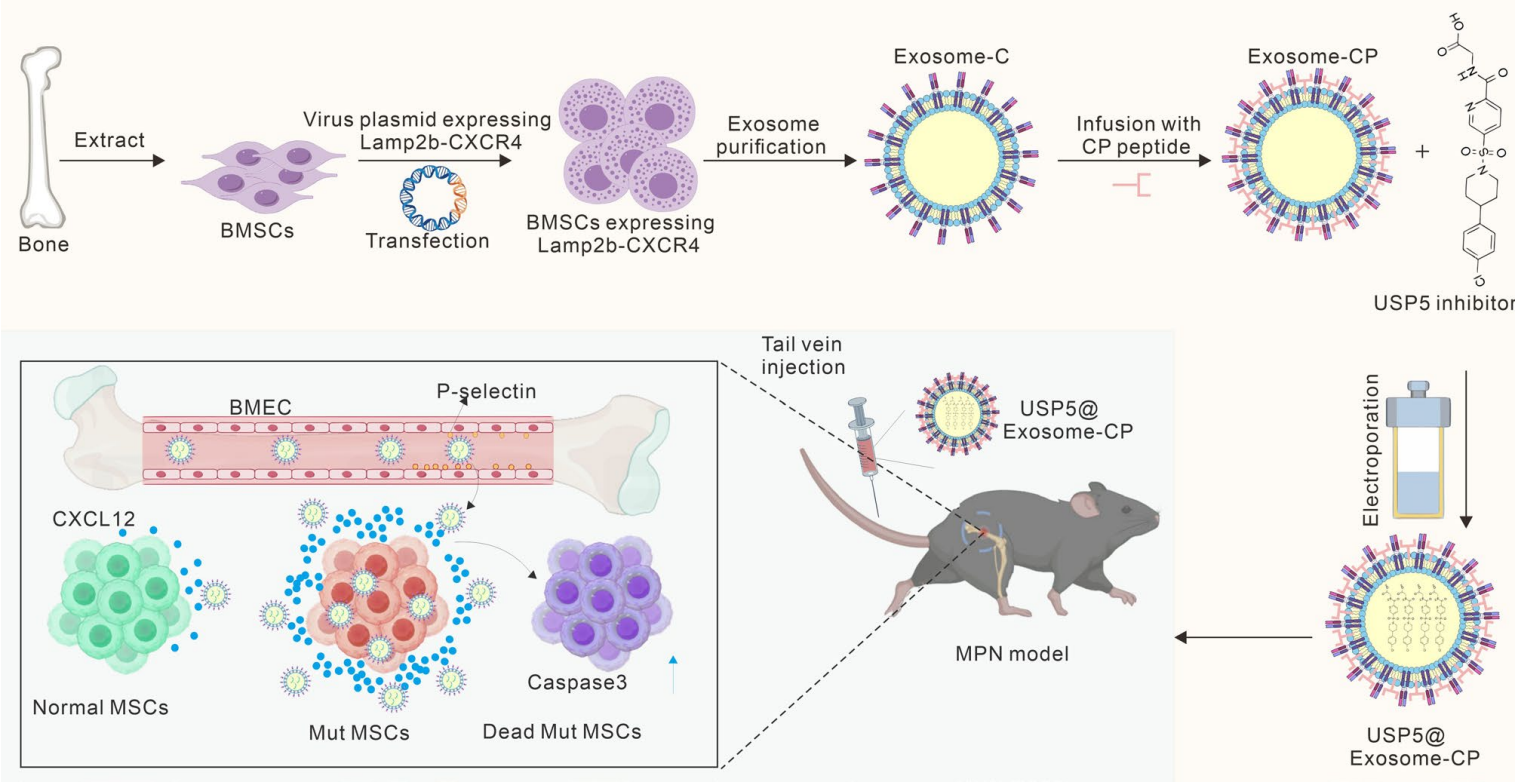
This study achieved MSCs-derived exosomes carrying CXCR4 proteins by means of genetic engineering, as well as adsorption of peptides targeting P-selectin through ligand-receptor-associated effects and loading of USP-5-IN-1, a drug that induces apoptosis in Mut-MSCs, via an electroporation strategy. The nanomedicine efficiently traversed the BMECs, targeted Mut-MSCs, and mediated their effective apoptosis, thus realizing the treatment of MPN model

To overcome the limitations of systemic drug delivery, we engineered MSC-derived exosomes (USP5@Exosome-CP) co-functionalized with CXCR4 and a P-selectin-targeting peptide (PSN). The dual-targeting design leverages the upregulated CXCL12 secretion in Mut-MSCs and the inflammatory overexpression of P-selectin on BM endothelial cells (BMEC) in MPN models. This strategy ensures efficient transendothelial transport and selective accumulation of exosomes in the BM niche. Loaded with USP5-IN-1, the engineered exosomes demonstrated sustained drug release, enhanced Mut-MSC targeting, and potent induction of apoptosis, culminating in prolonged survival of MPN model mice (Scheme 1). By integrating mechanistic insights into USP5-driven MPN progression with advanced nanocarrier engineering, this study provides a transformative therapeutic platform. Our approach not only validates USP5 as a biomarker and target but also pioneers the use of biomimetic exosomes for precision therapy in hematological malignancies, offering a blueprint for future applications in oncolog.

## Results

### Screening of deubiquitinating enzymes for regulating aberrant proliferation of MSCs

**Fig. 1:**
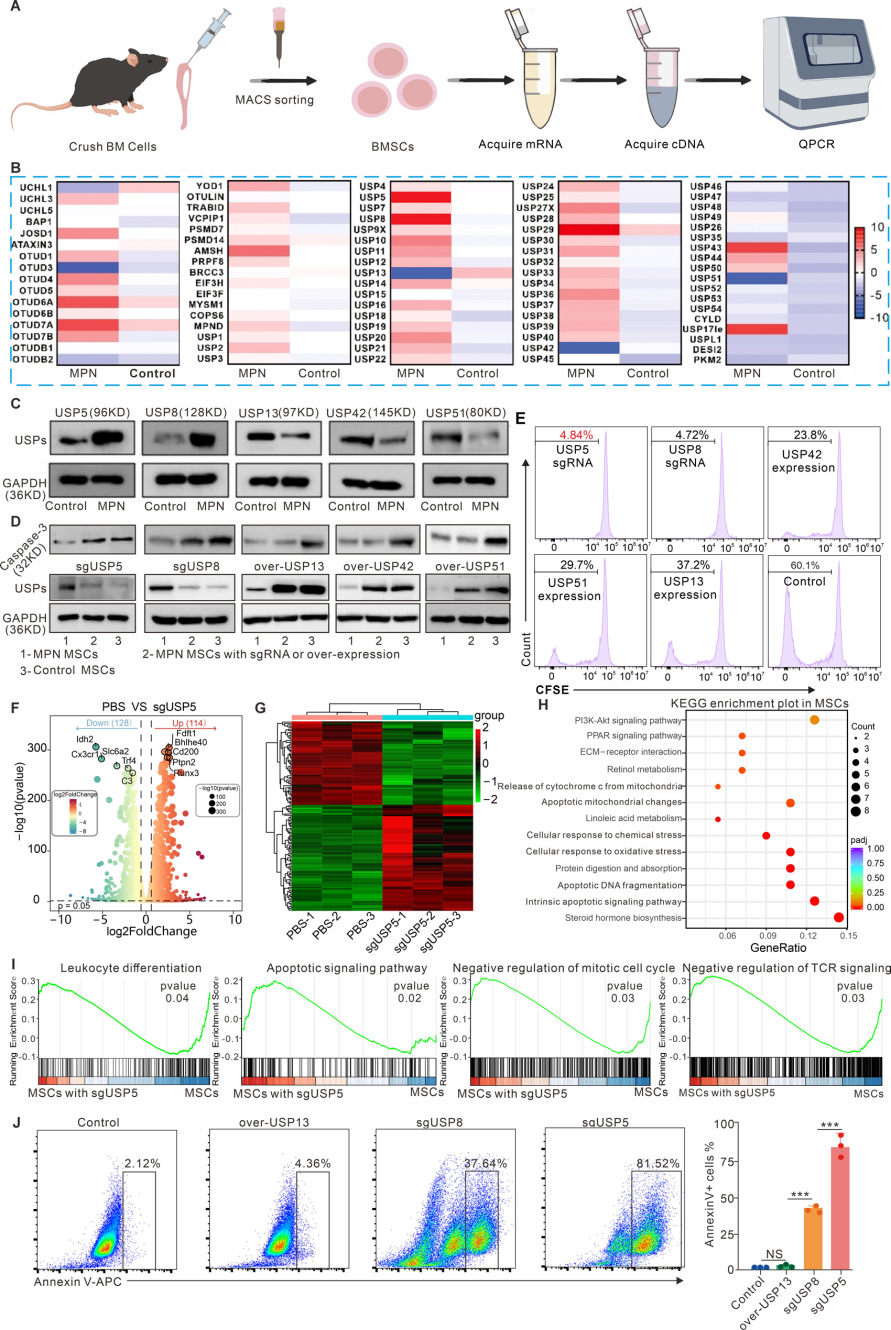
Identification of deubiquitinating enzymes for regulating the proliferative properties of MSCs obtained from MPN mouse model. (**A**) Schematic diagram of the experimental procedure for obtaining cDNA from mouse BM hematopoietic stem cells. (**B**) Differential expression statistics of the deubiquitinating enzyme family at the mRNA level in normal MSCs and in MSCs sorted from the BM of mice harboring a mutation in the *JAK2*^V617F^ gene. (**C**) Western blotting experiments to verify differences in protein expression levels of differentially expressed deubiquitinating enzymes screened from (**B**). (**D**) Western blotting experiments to verify the effect of knockdown or overexpression of differentially expressed deubiquitinating enzymes on caspase-3 expression. (**E**) Flow cytometry assay to verify the effect of overexpression or knockdown of differentially expressed deubiquitinating enzymes on the proliferative activity of MSCs derived from the MPN mouse model. (**F**) Volcano plot of differential gene expression in the PBS group and sgUSP5 group. (G) Heat map of clustering analysis of differential gene in sgUSP5 treated MSCs. (**H**) KEGG pathway enrichment analysis in sgUSP5 treated MSCs. (**I**) Enrichment analysis of GSEA gene set in sgUSP5 treated MSCs. (**J**) Representative flow cytometry plots and quantitative analysis of Annexin V positive cells after 24 h of different treatment in MSCs. Data are presented as the mean ± SD. *P < 0.05, **P < 0.01, ***P < 0.001, and NS: not significant. *N* = 3 experimental replicates

Overproliferation of hematopoietic stem cells leads to rapid progression of MPN, concurrently shortening the therapeutic window for patients [25]. Curtailing the clonal proliferation of hematopoietic stem cells in MPN and effectively controlling the disease’s progression are prerequisites for facilitating effective treatments. This study delves into the proliferation characteristics of MSCs carrying the *JAK2*^V617F^ mutation (Mut-MSCs), aiming to uncover deubiquitinating enzymes associated with the abnormal proliferation of these cells. Figure 1A illustrates the experimental workflow for screening deubiquitinating enzymes that are specifically overexpressed in Mut-MSCs with abnormal proliferation at the transcriptome level. We initially isolated MSCs from the BM of normal and *JAK2*^V617F^ mutation-bearing mice using a CD117 magnetic bead selection kit, and MSCs or Mut-MSCs occupied more than 95% of the isolated cells (Figure S1,** Supporting information**). Total mRNA was extracted, and complementary DNA (cDNA) was synthesized from normal MSCs or Mut-MSCs. Subsequently, we utilized our self-constructed primer library, encompassing 86 deubiquitinating enzymes (Supporting information Table 1), to identify enzymes with differential expression between normal MSCs and Mut-MSCs. Quantitative PCR QPCR results presented in Fig. 1B revealed that USP5 and USP8 were most highly expressed in Mut-MSCs compared to normal MSCs, while USP13, USP42, and USP51 were significantly downregulated. Considering the potential for post-translational modifications, we employed western blotting experiment to compare the expression of USP5, USP8, USP13, USP42, and USP51 in normal and Mut-MSCs. The results in Fig. 1C confirmed that the mRNA expression levels of differentially expressed deubiquitinating enzymes in normal and Mut-MSCs were consistent with their protein expression levels.

Given the correlation between cellular proliferation characteristics and the expression of apoptotic proteins, we compared the expression of caspase-3, a protein closely associated with apoptosis, to determine its correlation with the differentially expressed deubiquitinating enzymes. We overexpressed USP13, USP42, and USP51 in Mut-MSCs or knocked down the expression of USP5 and USP8, and then assessed caspase-3 expression in Mut-MSCs following these treatments. Western blotting results in Fig. 1D showed that Mut-MSCs expressed lower levels of caspase-3 protein compared to normal MSCs. Overexpression of USP13, USP42, and USP51 did not affect caspase-3 expression in Mut-MSCs, but knocking down USP5 or USP8 significantly enhanced Caspase-3 expression to levels comparable to those in normal MSCs. Additionally, we verified the proliferation of Mut-MSCs following these treatments. Flow cytometry results in Fig. 1E confirmed that while overexpression of USP13, USP42, and USP51 did not influence caspase-3 expression, it moderately inhibited the proliferation of Mut-MSCs. In contrast, knocking down USP5 or USP8 nearly abrogated MSC proliferation. These findings confirm that USP5 or USP8 can specifically regulate caspase-3 expression in Mut-MSCs and serve as effective targets to inhibit their proliferation. However, since the efficacy of USP8 has been reported in other studies [26], this research focuses on modulating USP5 activity to achieve effective treatment of MPN models.

To demonstrate the mechanisms of USP5 regulating the proliferation of Mut-MSCs, we performed bulk RNA sequencing in Mut-MSCs after treatment with or without knock outing of USP5 gene. The volcano plot of differentially expressed genes and clustering results were shown in the Fig. 1F-I. After treatment with knock outing of USP5 gene, expression levels of oncogenes such as *Idh2* and *Slc6a2* were significantly downregulated, while levels of tumor suppressor genes such as *Ptpn2* and *Runx3* were significantly upregulated (Fig. 1F-G). KEGG enrichment analysis showed that pathways related to apoptosis were markedly upregulated (Fig. 1H). Particularly, GESA pathway analyzes revealed that cell differentiation, apoptosis, negative regulation of cell cycle and negative regulation of TOR signaling were enriched in knock outing of USP5 gene group (Fig. 1I), which was in line with our flow cytometry results (Fig. 1J). We hypothesize that USP5 may deubiquitinate and stabilize anti-apoptotic proteins (e.g., XIAP), leading to Caspase-3 suppression, though direct targets require future investigation [26]. Overall, these data indicate that knockdown of USP5 gene in Mut-MSCs induces apoptosis and differentiation and reduces cell cycle related and other oncogenic pathways.

USP5-IN-1 was reported to efficiently inhibit USP5 activity [27], therefore, we used this inhibitor to verify the killing effect on Mut-MSCs or MSCs. The results of Figure S2 (Supporting information) showed that low concentrations of USP5-IN-1 exhibited a higher killing effect on Mut-MSCs and a weaker killing effect on MSCs, but if knockdown of USP5 expression in Mut-MSCs, it was able to reverse Mut-MSCs sensitivity, demonstrating that USP5-IN-1 can specifically inhibit the function of USP5 protein. In addition, we also tested the effects of USP5-IN-1 or knockdown of USP5 on Mut-MSCs-mediated MPN model. To construct the MPN model, we obtained Mut-MSCs from the BM of *JAK2*^V617F^ mutated mice and constructed luciferase-expressing Mut-MSCs by retroviral system, and subsequently injected these Mut-MSCs into normal C57 mice that had undergone BM cleaning with X-ray via tail vein. The results in Figure S3 (**Supporting information**) showed that USP5 knock down or USP5-IN-1 therapy could effectively inhibit the generation of MPN models, whereas Mut-MSCs alone were able to rapidly form MPN models. The above results confirm the importance of USP5 in mediating the formation of MPN models in MSCs.

### Bioinformatic analysis of USP5 expression correlates with disease progression in acute myeloid leukemia patients (AML)

**Fig. 2:**
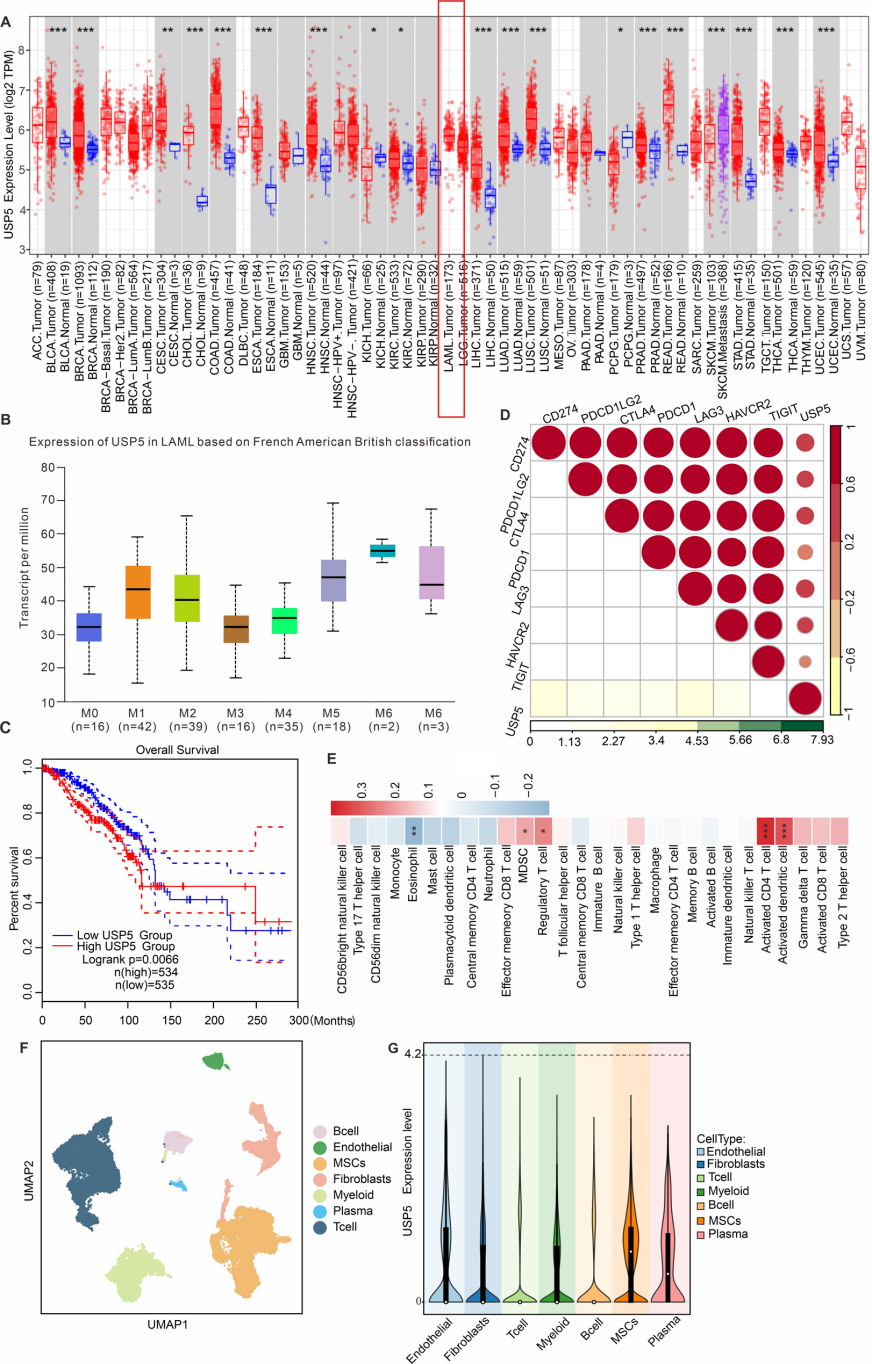
Bioinformatic analysis of USP5 expression correlates with disease progression in acute myeloid leukemia patients (AML). (**A**) Analysis of USP5 mRNA expression in different cancer species based on TCGA database. (**B**) Analysis of USP5 mRNA expression in AML patients with different disease stage. (**C**) Survival analysis of AML patients with high expression of USP5 versus low expression of USP5. (**D**) Analysis of USP5 expression in tumor tissue in relation to the expression of different immunoregulatory proteins in TCGA database. (**E**) Analysis of USP5 expression in AML BM in relation to the infiltration of different immune cells in TCGA database. (**F**) Unsupervised clustering algorithm to analyse different cell subpopulations in single-cell data from GSEA AML patients. (**G**) Analysis of different cell subpopulations expressing USP5 in AML patients

To verify whether USP5 is associated with survival and the immune microenvironment in MPN patients, we analyzed USP5 expression using a database of AML patients associated with the MPN disease model. Therefore, we first evaluated the expression of USP5 in AML from mRNA level. The results of Fig. 2A showed that the mRNA of USP5 was highly expressed in AML patient, and the high expression of USP5 was present at different stages of AML progression (Fig. 2B). This demonstrates that the high expression of USP5 is closely related to AML disease progression. Meanwhile, ALM patients with high USP5 expression had significantly lower survival than AML patients with low EIF2S2 expression (Fig. 2C). In addition, we also simultaneously analyzed the correlation of USP5 expression with the expression of immunomodulatory molecules and immune cell infiltration (Fig. 2D), and the results showed that highly USP5 expression promotes high expression of relevant immunosuppressive molecules such as PD-L1, PD-1, CTLA4, LAG3 and TIGIT in the tumor microenvironment, which in turn limits anti-tumor immunity. Furthermore, highly USP5 expression in AML patient is also significantly positively correlated with a large infiltration of immunosuppressive cells, such as myeloid immunosuppressive cells and regulatory T cells (Fig. 2E). By analyzing the single-cell database of AML (Fig. 2F-G), we found that USP5 was predominantly expressed in MSCs with high expression of CD34 and CD117. These results demonstrated that USP5 gene mainly promote the development of AML. Although AML is distinct from MPN, we analyzed USP5 in AML due to (1) shared molecular features in bone marrow dysfunction, and (2) limited availability of MPN patient databases with transcriptomic data. Our findings suggest USP5’s broader role in myeloid malignancies, warranting future validation in MPN cohorts.

### Preparation and characterization of USP5@exosome-CP

Although the genetic silencing of USP5 in Mut-MSCs can efficiently inhibit their abnormal proliferation, in vivo gene editing of target cells remains challenging. USP5-IN-1 has been reported to potently inhibit USP5 activity at low concentrations. However, its hydrophobic nature limits its in vivo application and clinical translation. Additionally, conventional in vivo drug administration of a single agent could not ensure effective BM infiltration and targeted regulation of Mut-MSCs. To address these challenges, some studies have focused on constructing bone marrow homing nanocarriers for targeting the bone marrow microenvironment [13, 15], where we propose to employ engineered stem cell-derived exosomes (MSCs) loaded with small molecule drugs such as USP5-IN-1 to achieve efficient in vivo targeting of BM Mut-MSCs.

**Fig. 3:**
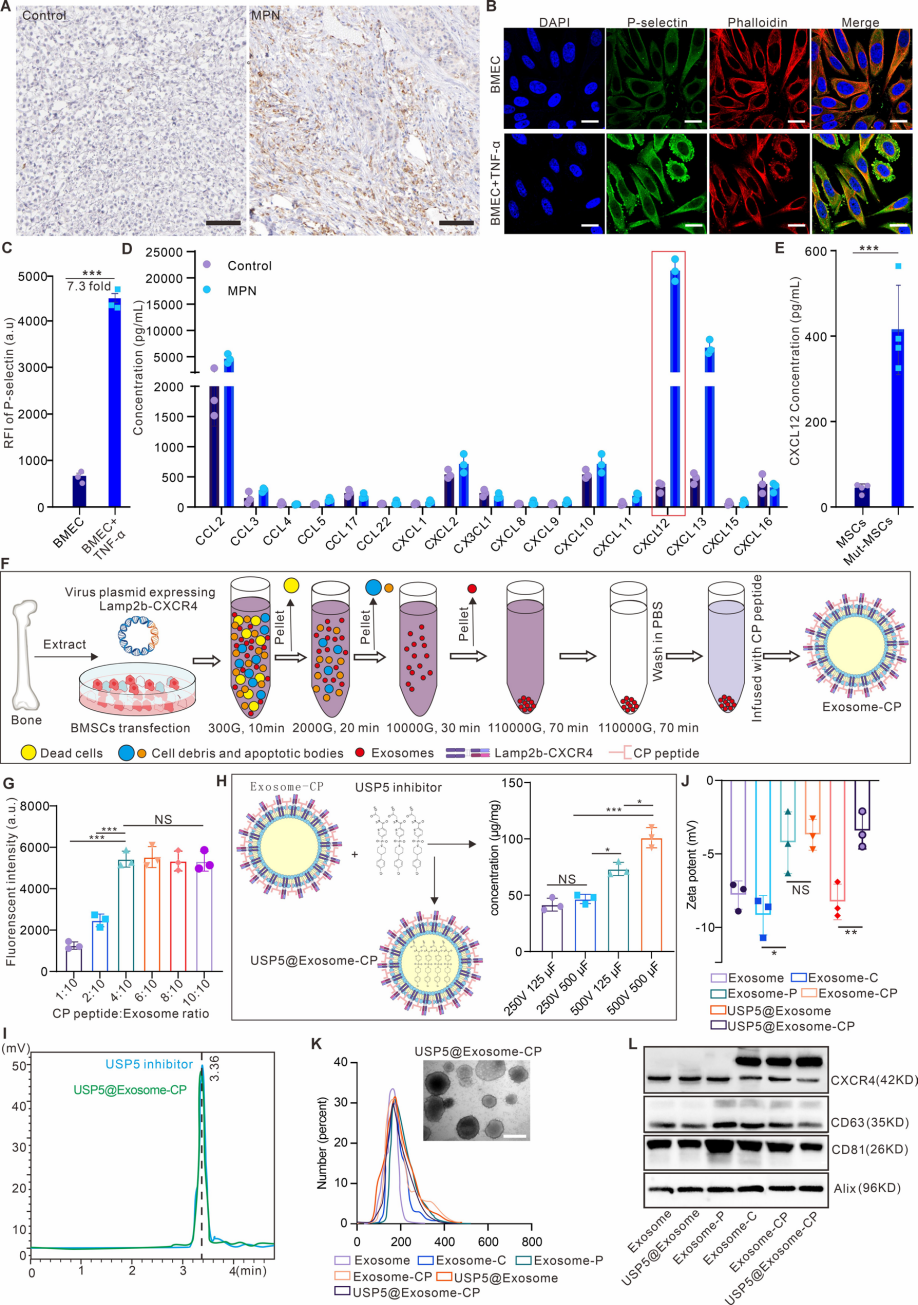
Preparation and characterization of USP5@exosome-CP. (**A**) Immunohistochemical assessment of P-selectin expression in bone marrow of MPN model mice. Scale bar is 100 μm. (**B**) Confocal imaging to assess bone marrow-derived vascular endothelial cells (BMEC) in inflammatory-stimulated high expression of P-selectin. Scale bar is 10 μm. (**C**) Flow cytometry to statistic the relative fluorescent intensity (RFI) of P-selectin in BMEC or BMEC infused with TNF-α. (**D**) Multi-cytokine detection kit to characterize the differences in the expression of different chemokines in the bone marrow microenvironment between MPN and normal mice. *N* = 3 experimental replicates. (**E**) Elisa assay to detect differences in CXCL12 secretion between normal MSCs and Mut-MSCs. *N* = 4 experimental replicates. (**F**) Schematic of Exosome-CP preparation. (**G**) Statistics of fluorescence intensity of engineered exosomes incubated with different mass ratios of FITC-labeled fusion peptide CP05-PSN (CP) for 24 h by using flow cytometry (The quality of the engineered exosomes remains unchanged, and the optimal amount of CP that can be loaded into the engineered exosomes is determined by adjusting the mass ratio of CP to engineered exosomes during incubation) (*N* = 3). (**H**) Statistics on the concentration of engineered exosomes loaded with USP5 inhibitor (USP5-IN-1) under different electrical stimulation conditions (*n* = 3). (**I**) HPLC to be used to identify the drug loading characteristics of USP5@Exosome-CP. (**J**) Statistics on zeta potential of different indicated engineered exosomes (*n* = 3), including Exosome, Exosome containing CXCR4 (Exosome-C), Exosome cotaining CP05-PSN (Exosome-P), Exosome containing CXCR4 and CP05-PSN (Exosome-CP), Exosome containing USP5-IN-1 (USP5@Exosome) and Exosome containing USP5-IN-1, CXCR4 and CP05-PSN (USP5@Exosome-CP). (**K**) Statistics on the range of particle size distribution of indicated engineered exosomes by using DLS and TEM (scale bar is 200 nm). (**L**) Validation of different types of exosomes expressing universal exosome markers or CXCR4/Lamp2b-CXCR4 by western bloting. Data are presented as the mean ± SD. *P < 0.05,**P < 0.01, ***P < 0.001, and NS: not significant

To facilitate exosome targeting to BM-derived MSCs (BMSCs), we first characterized the BM microenvironment. Exosomes need to cross the barrier of BM vascular endothelial cells (BMEC) before targeting BMSCs, whereas the exosome itself is weakly characterized for crossing vascular endothelial cells. To promote the specific targeting of exosome to BMEC, we first analyzed the expression of p-selectin in BMEC, as p-selectin has been reported to be expressed predominantly on the inflammatory vascular endothelium and most of the immune cells can cross the vascular endothelium via P-selectin [24, 28]. Immunohistochemical results (Fig. 3A) showed that the expression of p-selectin in BM from the MPN model was much higher than that in BM from normal mice, and the confocal imaging results (Fig. 3B-C) also showed that the expression of p-selectin in BMEC from the MPN model was much higher than that in BMEC from the normal mouse model. The above results confirmed that p-selectin can serve as a good target for exosomes to cross the vascular endothelial cells. Moreover, to further promote the targeting properties of exosomes to mutant MSCs, we analyzed the expression of different chemokines in BMEC from MPN model. The results of multi-cytokine assay (Fig. 3D) showed that the expression of CXCL12 was the highest in the BM of MPN and was significantly different from that of normal BM. The results of Elisa assay (Fig. 3E) further confirmed that the secretion of CXCL12 in Mut-MSCs was also significantly higher than that in the MSCs cells, and the above results suggested that we could target Mut-MSCs of MPN model through the interactions such as CXCR4/CXCL12.

To ensure that CXCR4 can be efficiently secreted into exosomes, we first express a fusion protein, Lamp2b-CXCR4, in MSCs derived from normal mouse BM (Figure S4,** Supporting information**), which is actively secreted into exosomes, thereby equipping MSC-derived exosomes with the CXCR4 protein. In addition, we embedded the fusion peptide CP05-PSN (peptide sequence is CRHSQMTVTSRLGGGGSDAEWVDVS), which can target p-selectin, in the exosomes loaded with CXCR4 protein (CP05 is a peptide targeting CD63 protein on exosomes [29], and PSN can target p-selectin, and auto-loading of the fusion peptide on exosomes can be facilitated by linker linking P-selectin targeting motif to CP05, such as GGGGS). The schematic diagram of exosomes extraction was in depicted in Fig. 3F.

To verify how much CP05-PSN (CP, a peptide fusion of CP05 and P-selectin targeting motif) peptide can be effectively loaded on the exosomes outer surface, we incubated different concentrations of CP peptide modified with FITC dye with equal protein concentration of exosomes (Exosome-CP). The statistics results in Fig. 3G by using flow cytometry showed that the fluorescence intensity of exosomes was saturated when the ratio of the protein concentration of CP peptide to exosomes was 2:5. In addition, to determine the stability of CP peptide bound to exosomes, we incubated it with 10% mouse plasma for different times, and the results of naive SDS-PAGE gels (Figure S5,** Supporting information**) showed that the CP peptide was able to be stably maintained on the surface of the Exosome-CP for about 36 h.

To verify the drug loading ability of Exosome-CP, we also explored the parameters of the electroporator for the exosomes to be effectively loaded with USP5-IN-1, and the results of Fig. 3H showed that when the voltage parameter of the electroporator was set to 500 V, and the capacitance parameter was 500µF, the exosomes could be loaded with the largest amount of USP5-IN-1 molecular. The HPLC results (Fig. 3I) confirmed that the peak times of the small molecules extracted from USP5@Exosome-CP coincided with those of free USP5-IN-1, proving that USP5@Exosome-CP indeed contains a large amount of USP5-IN-1, and does not change its chemical properties. To verify the stability of USP5-IN-1 in USP5@Exosome-CP, we also used HPLC to characterize the amount of USP5-IN-1 remaining in USP5@Exosome-CP after incubation with 10% mouse plasma for different times. The results (Figure S6,** Supporting information**) showed that the half-life (t1/2) of USP5-IN-1 in USP5@Exosome-CP under physiological conditions (10% mouse serum) was approximately 48 h, with ~ 60% remaining after 72 h, demonstrating the hydrophobicity of USP5@Exosome-CP-loaded stability of the hydrophobic drug. We also measured the zata potential and particle size of exosomes processed in various ways and found that the loading of peptide or drug did not significantly affect the zata potential of exosomes (Fig. 3J), whereas the particle size of exosomes loaded with drug increased to a certain extent (Fig. 3K). The results in Figure S7 (Supporting information) showed that the loading of CP peptide and USP5-IN-1 agents did not influence the structure of the exosomes.

Western blot was used to characterize whether CXCR4 is efficiently loaded into exosomes. Figure 3L demonstrated that all the exosomes were rich in exosome-associated proteins such as CD63 and CD81, whose expression was not influenced by encapsulation of CP peptide and USP5-IN-1 agents, and CXCR4 was also efficiently loaded into engineered exosomes. Thus, the exosomes that we extracted had the typical characteristics of small extracellular vesicles. In summary, these data confirmed the successful construction of a biomimetic nanocarrier with optimized drug loading. The simple synthesis strategy used here maximizes drug delivery and target motif loading, demonstrating the potential of engineered MSCs-derived exosomes for further nano-drug development.

### Validation of USP5@Exosome-CP targeting inflammatory BM-derived endothelial cells and Mut-MSCs

**Fig. 4:**
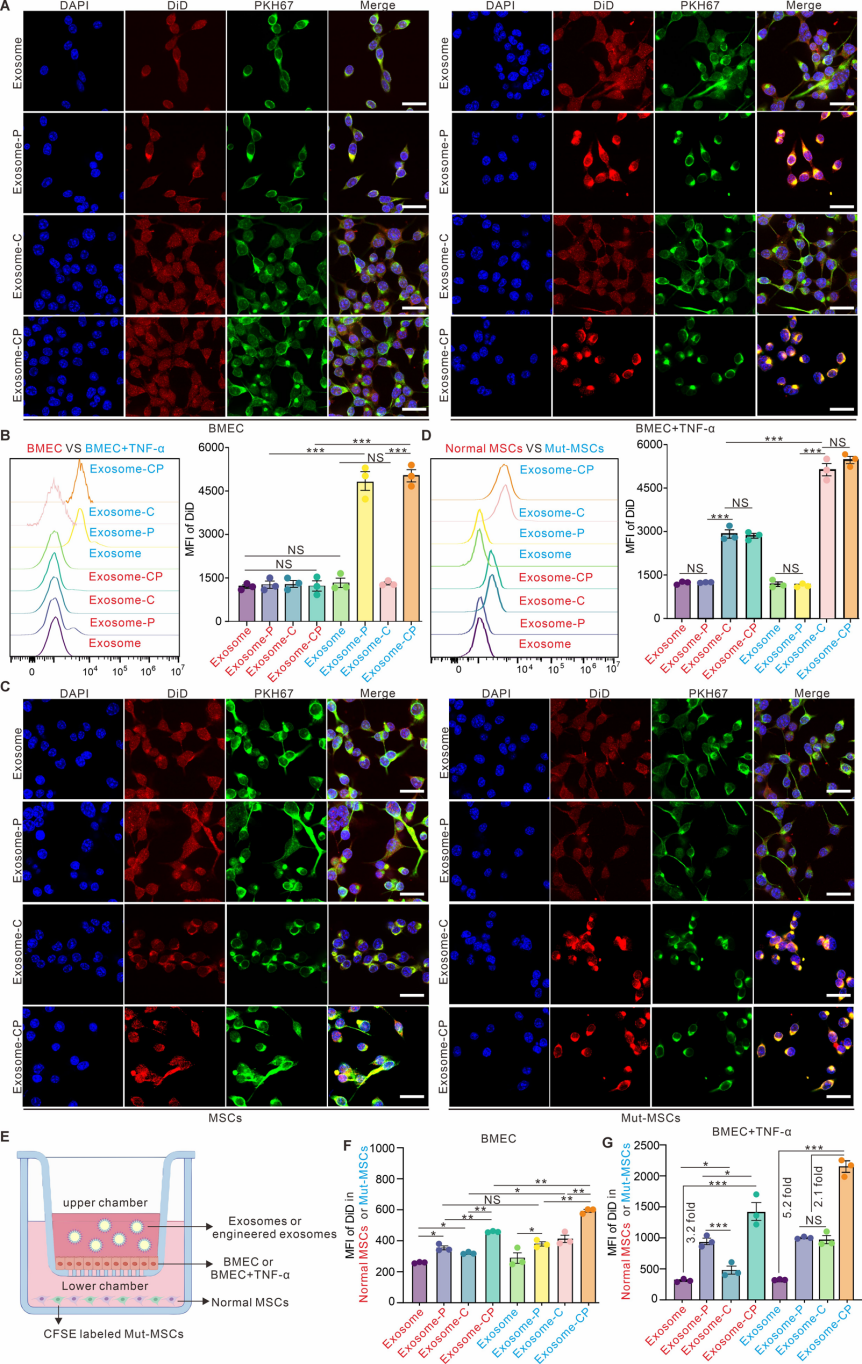
Characterization of different engineered exosomes targeting inflammatory endothelial cells and BMSCs. (**A**) Confocal imaging characterization of different engineered exosomes targeting normal BMECs and inflammatory BMECs (infused with TNF-α). Scale bar is 20 μm. (**B**) Flow cytometry characterization of different engineered exosomes targeting normal BMEC and inflammatory BMEC (infused with TNF-α). (**C**) Confocal imaging characterization of different engineered exosomes targeting normal MSCs and Mut-MSCs. Scale bar is 20 μm. (**D**) Flow cytometry characterization of different engineered exosomes targeting normal MSCs and Mut-MSCs. (**E**) Schematic diagram of the Transwell model validating different engineered exosomes across normal BMEC or inflammatory BMEC. (**F**) Flow cytometry statistics on the properties of different engineered exosomes targeting normal MSCs and Mut-MSCs after crossing normal BMEC. (**G**) Flow cytometry statistics on the properties of different engineered exosomes targeting normal MSCs and Mut-MSCs after crossing an inflammatory BMEC. Data are presented as the mean ± SD. *P < 0.05,**P < 0.01, ***P < 0.001, and NS: not significant. *N* = 3 experimental replicates

In order to validate the specific targeting capacity of engineered exosomes (with single or dual targeting properties) towards inflammatory BM-derived endothelial cells (BMEC) and MSCs. Systematic evaluation of targeting characteristics was performed using confocal microscopy and flow cytometry for various engineered exosomes (Exosome, Exosome-CP, Exosome-C, Exosome-P), with a focus on their efficiency in targeting normal/inflammatory BMECs and Mut-MSCs. As demonstrated in Fig. 4A, confocal imaging revealed low uptake efficiency of both P-selectin-targeting peptide modified and CXCR4-functionalized exosomes by normal BMECs. However, P-selectin modified exosomes (Exosome-P) exhibited significantly enhanced endocytosis in inflammatory BMECs. Quantitative flow cytometry analysis (Fig. 4B) further confirmed this observation, highlighting the critical role of P-selectin-targeting peptides in mediating exosome transendothelial transport across the inflammatory vascular barrier in MPN models. We also elucidated the mechanism of CXCR4 modules in targeting CXCL12-hypersecreting Mut-MSCs, various engineered exosomes were co-cultured with normal MSCs and Mut-MSCs for 24 h. Figure 4C-D demonstrated that CXCR4-modified exosomes (Exosome-C) significantly enhanced targeting efficiency towards MSCs, with particular specificity for Mut-MSCs, confirming the pivotal role of the CXCR4/CXCL12 axis in Mut-MSC targeting.

Building upon these findings, we established a Transwell model (Fig. 4E) to simulate exosome transendothelial delivery. The upper chamber contained normal/inflammatory BMEC monolayers, while the lower chamber housed a co-culture system of CFSE-labeled Mut-MSCs and normal MSCs. Following 24-hour incubation with DiD-labeled exosomes in the upper chamber, flow cytometric analysis of lower chamber cells (Fig. 4F-G) showed that dual-modified exosomes (Exosome-CP) demonstrated superior targeting efficiency towards Mut-MSCs after traversing inflammatory BMEC layers, with mean fluorescence intensity (MFI) approximately 5-fold higher than those of Exosome-C and Exosome-P. This enhanced performance suggests that Exosome-CP’s superior penetrability and targeting specificity may originate from the selective binding capacity of its surface P-selectin-targeting peptide within inflammatory microenvironments. Collectively, Exosome-CP exhibits remarkable advantages in targeting inflammatory endothelia and Mut-MSCs, providing a novel delivery platform for precision therapy in inflammation-related diseases and MPN models. In addition, to confirm whether Exosome-CP carrying USP5-IN-1 could efficiently induce apoptosis in Mut-MSCs, we used engineered exosomes of different designated treatment groups to co-incubate with Mut-MSCs in vitro for 24 h, and then observed the death ratio of Mut-MSCs in different groups by staining PI dye. The flow cytometry statistical results in Figure S8 (Supporting information) confirmed that USP5@Exosome-CP was able to induce significant death of Mut-MSCs, which was consistent with the effect of incubating USP5-IN-1 alone, but much more effective than that of Exosome-CP as well as exosomes. The above results confirmed that USP5@Exosome-CP had both targeting and therapeutic effect.

### Biodistribution of USP5@exosome-CP

**Fig. 5:**
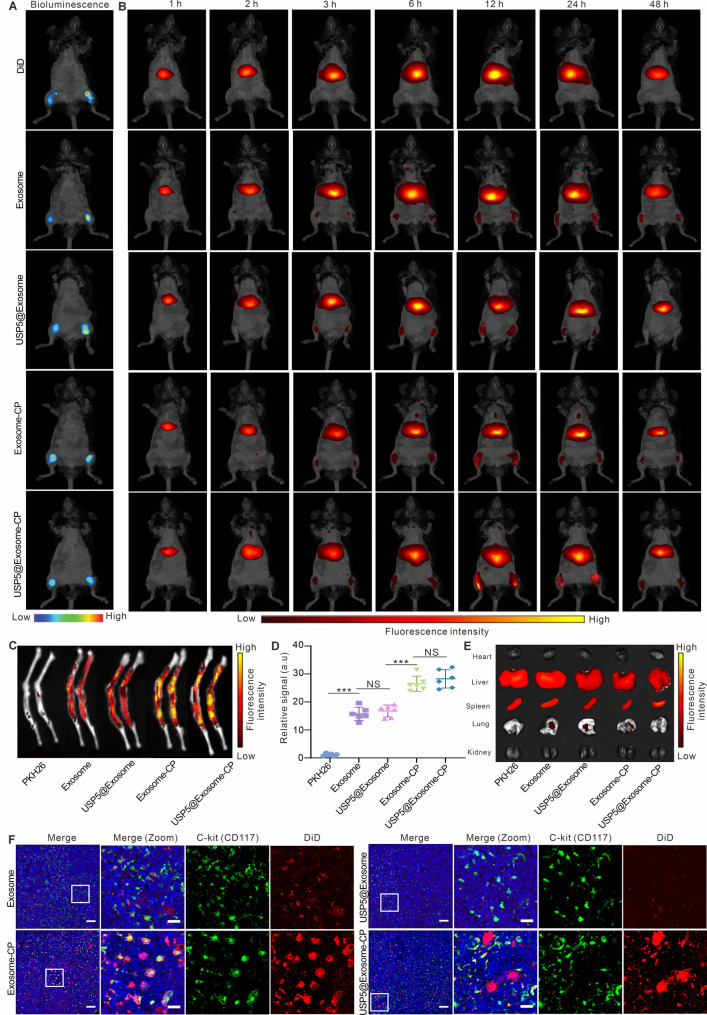
Biodistribution and BM-targeting ability of USP5@Exosome-CP in vivo. (**A**) Whole body imaging to verify the successful MPN modeling in different treatment group. (**B**) Radiant efficiency-based fluorescence imaging of C57BL/6J mice at various time points post-intravenous injection of DiD labeled Exosome, DiD labeled USP5@Exosome, DiD labeled Exosome-CP and DiD labeled USP5@Exosome-CP. There were *N* = 3 mice per group. The BMs are indicated by blue ovals. (**C**) Fluorescence images of bone tissues from mice injected with indicated treatment. (**D**) Region-of-interest analysis of fluorescent intensities from bone tissues. *N* = 6 (three mice with each mouse per group captured twice). (**E**) The distribution of indicated treatment in various organs. (**F**) Immunofluorescence was used to assess the ability of exosomes from different designated treatment groups to target BMSCs in the MPN model. DAPI (blue) was used to stain nuclei, C-kit (Green) was used to stain MSCs, and DiD was used to trace exosomes. Scale bar is 100 μm and the scale bar of the zoom image is 30 μm. The units of the colour bars in (**A**), (**B**) and (**D**) are ps^–1^ cm^–2^ sr^–1^/µW cm^–2^. Data are presented as the mean ± SD. *P < 0.05,**P < 0.01, ***P < 0.001, and NS: not significant

To assess the BM-homing capability of the USP5@Exosome-CP nanosystem, different engineered exosomes of equal protein concentration after DiD staining were injected via tail vein into MPN model mice carrying the *JAK2*^V617F^ mutation. The bioluminescence imaging results in Fig. 5A showed that we successfully constructed a luciferase-expressing MPN model. We performed fluorescence imaging of mice injected with different specified treatments at different time points, and the results in Fig. 5B showed that the unengineered exosomes themselves had the ability to target the BM, and the Exosome-CP carrying CXCR4 and CP peptide was able to further promote their accumulation in the BM of mice. In addition, carrying USP5-IN-1 did not affect the targeting properties of engineered exosomes in BM. The fluorescence signal of USP5@Exosome-CP or Exosome-CP appeared at 3 h and peaked at 12 h post-injection. By contrast, the DiD groups underscored the minimal BM-homing capabilities. The fluorescence signals of dissected bone tissue also revealed significantly stronger fluorescence intensity of USP5@Exosome-CP or Exosome-CP in comparison with exosomes or USP5@Exosome, with no detectable fluorescence of the DiD groups. Specifically, USP5@Exosome-CP exhibited a 2.2-fold increase in fluorescence intensity compared to unmodified Exosome (*P* < 0.001) and a 2.1-fold increase compared to USP5@Exosome (*P* < 0.01) (Fig. 5C-D). In addition, fluorescence imaging maps of the major organs of mice in the different designated treatment groups showed that the engineered or unengineered exosomes accumulated mainly in the liver and spleen, with lower levels of accumulation in other organs (Fig. 5E). To further evaluate the targeting properties of USP5@Exosome-CP on in vivo Mut-MSCs, we observed the BM of different groups of mice using immunofluorescence. The results in Fig. 5F showed that Exosome and USP5@Exosome infiltrated into the BM with weak fluorescence intensity and co-localized weakly with Mut-MSCs cells (c-kit^+^), whereas Exosome-CP and USP5@Exosome-CP infiltrated into the BM with significantly enhanced ability and had strong fluorescence co-localization with Mut-MSCs. The above results confirmed that USP5@Exosome-CP could efficiently traverse BMEC in vivo and could highly target Mut-MSCs cells in the BM.

### Anti-MPN effects of USP5@Exosome-CP

**Fig. 6:**
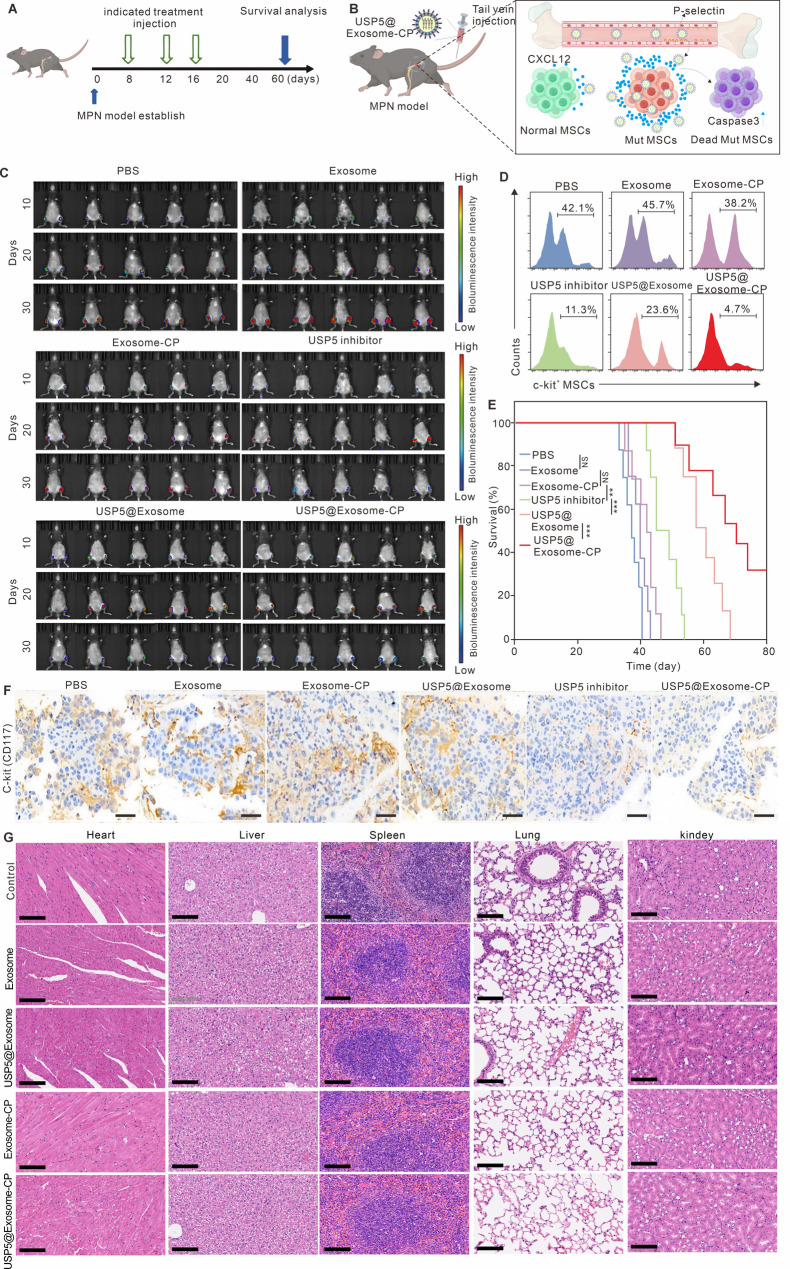
Anti-MPN effects of USP5@exosome-CP in vivo. (**A**) Schematic of the MPN modeling and treatment protocol. There were *N* = 10 mice per group. The therapeutic concentration used in in vivo experiments was 5 mg/kg USP5-IN-1 (equivalent to 50 mg/kg exosome per mouse). (**B**) Schematic illustration of the therapeutic process of USP5@exosome-CP. (**C**) Bioluminescence images of mice treated with groups. The units of the colour bars are counts. (**D**) Flow cytometry analysis of MSCs in BM. (**E**) Survival curves of MPN mouse model treated with different formulations. the survival statistical significance was analysed via a log-rank (Mantel-Cox) test. (**F**) Immunohistochemical assessment of the expression intensity of c-kit^+^ positive cells in bone marrow in different designated treatment groups. Scale bar is 200 μm. (**G**) Representative histological examinations of H&E stained organs from mice receiving indicated treatment. Scale bar is 200 μm

The therapeutic efficacy of USP5@Exosome-CP was assayed in MPN mice model (Fig. 6A), and the therapeutic process is depicted in Fig. 6B. The therapeutic concentration used in in vivo experiments was 5 mg/kg USP5-IN-1 (equivalent to 50 mg/kg exosome particles per mouse). The bioluminescence signals of the different designated treatment groups were obtained on days 10, 20 and 30. The results in Fig. 6C showed that tail vein injection of exosome or Exosome-CP did not demonstrate a therapeutic advantage in the MPN model, whereas USP5-IN-1 administered intraperitoneally alone was able to inhibit the development of MPN to a certain extent, but the effect was not significant, which was mainly related to its effective concentration of the drug in the BM, whereas USP5@Exosome was able to inhibit the MPN development further in comparison with the administration of USP5-IN-1 alone development, and USP5@Exosome-CP with targeted homing properties demonstrated the highest degree of therapeutic effect on MPN. Figure 6D also demonstrates that the mice treated with USP5@Exosome-CP manifested more potent eradication of Mut-MSCs compared with other groups. The survival rate after 80 days in mice receiving USP5@Exosome-CP was approximately 30% (Fig. 6E). These findings indicated that USP5@Exosome-CP may have a positive impact on BM function and contribute to the recovery of MPN model. To assess the biosafety of USP5@Exosome-CP, H&E staining was used to evaluate the potential toxicities in different designated treatment groups. Figure 6F shows immunohistochemical staining of c-kit^+^ cells in bone marrow, revealing reduced Mut-MSCs in USP5@Exosome-CP-treated mice. Figure 6G shows H&E-stained organs without pathological changes. These results confirm that USP5@Exosome-CP can serve as a superior therapeutic strategy for MPN models.

## Discussion

In this study, we have successfully identified USP5 as a critical regulator of MSCs proliferation in MPN driven by the *JAK2*^V617F^ mutation. Our findings demonstrate that USP5 overexpression in MSCs contributes to the aberrant proliferation observed in MPN by modulating the apoptosis pathway, specifically through the regulation of Caspase-3. This discovery underscores the importance of targeting USP5 as a therapeutic strategy for MPN, highlighting its potential as a novel biomarker and therapeutic target.

We have further developed an innovative approach to deliver USP5 inhibitors directly to the BM microenvironment using engineered stem cell-derived exosomes. Unlike conventional therapies such as hydroxyurea and ruxolitinib, which often face challenges of systemic toxicity and limited bone marrow penetration, our engineered exosomes enable precise delivery to the bone marrow niche, thereby enhancing efficacy while minimizing off-target effects. These exosomes, co-expressing CXCR4 and a P-selectin-targeting peptide, efficiently home to BM MSCs, overcoming the challenges of traditional drug delivery methods. The encapsulation of USP5-IN-1 within these exosomes ensures sustained release and effective inhibition of USP5 activity, leading to significant suppression of MSCs proliferation and induction of apoptosis. While our engineered exosomes show promise, scaling production and ensuring batch-to-batch consistency remain challenges for clinical translation. Future studies will focus on optimizing GMP-compliant manufacturing processes [20].

Our findings establish USP5 as a critical therapeutic target in MPN, with dual-targeted exosomes offering a promising strategy for clinical translation by overcoming the limitations of current therapies. While USP5-IN-1 at high concentrations (> 5 µM) induced death in normal MSCs, our targeted delivery system (USP5@Exosome-CP) achieved selective killing of Mut-MSCs at therapeutic doses (1 µM) without affecting normal MSCs (Fig S2, S8). This specificity is attributed to: (1) higher USP5 dependency in Mut-MSCs, and (2) exosome-mediated targeting reducing systemic exposure. The use of engineered exosomes as nanocarriers represents a significant advancement in targeted drug delivery, potentially improving treatment efficacy and reducing systemic toxicity [30–32]. This approach could be further explored for other hematological malignancies and solid tumors, where precise targeting of the tumor microenvironment is crucial for effective therapy. Overall, our research opens new avenues for the development of personalized and targeted therapies for MPN, contributing to the broader field of cancer research and treatment.

## Materilas and method

### Materials

Fetal Bovine Serum (FBS) was obtained from Zhejiang Tianhang Biotechnology Co., Ltd. (Huzhou, China). Plasmocin was bought from InvivoGene (Toulouse, France) and penicillin/streptomycin was obtained from Biosharp (Hefei, China). Sterile 1×phosphate bufered saline was purchased from Gibco Life Technologies, Inc. (Grand Island, NY, USA). CXCL12 chemokine were purchased from Biolegend (San Diego, CA, USA). USP5-IN-1 was bought from MCE (cat number: HY-139979). Acetonitrile, methanol and chloroform for High Performance Liquid Chromatography (HPLC) were all purchased from Termo Fisher Scientifc (Waltham, MA, USA) and their purity was more than 99%. The fluorescence dye DiD or CFSE (5,6- carboxyfluorescein diacetate, succinimidyl ester) were obtained from MedChemExpress (NJ, USA). Radioimmunoprecipitation assay buffer and the inhibitors of protease and phosphatase were obtained from Beyotime (Shanghai, China). For western blot, primary antibodies CD63, CD81 and Alix were obtained from Proteintech Group, Inc. (Chicago, IL, USA). Secondary antibodies goat anti-mouse IgG H&L-HRP conjugated and goat anti-rabbit IgG H&L-HRP conjugated were bought from Abcam (Cambridge, UK). All the antibodies for fow cytometry and immunofluorescence were bought from Biolegend (San Diego, CA, USA). CP05-PSN peptide (CRHSQMTVTSRLGGGSDAEWVDVS) or FITC labeled CP05-PSN peptide was purchased from Shanghai Apeptide Co., Ltd. (Shanghai, China).

### Cell lines

Mouse bone marrow-derived stem were generated as previously described [33]. Murine MSCs were purchased from Pro-cell (Cat number # CP-M131) and cultured in Complete medium (specifically designed for mouse MSCs, purchased from Pro-cell) containing 10% (v/v) FBS and 1% (v/v) penicillin/streptomycin. All the mediums were added with 10% (v/v) FBS and 100 µg mL^− 1^ penicillin/streptomycin.

### BMECs isolation

The BMECs of mice were acquired using a previously described approach [34]. In brief, the epiphyses of bones were removed and the marrow was flushed out and cultured overnight with Dulbecco’s modified Eagle’s medium (DMEM) supplemented with 20% FBS. Then, the floating cells were discarded. The adhered cells were digested with trypsinization and selected using CD31 microbeads to isolate BMECs. The obtained BMECs were grown in a culture flask pretreated with rat-tail collagen type 1. The BMECs were cultured with a ECM kit (Oricell) that contained growth factors for ECs. FCM and tube formation assays were conducted for the characterization of the isolated BMECs.

### Reverse transcription quantitative PCR (RT-qPCR) analysis

Total RNAs were extracted using the TRIzol reagent (Invitrogen), and reverse transcription reactions were performed using the PrimeScript RT reagent kit (TaKaRa, Cat. No. RR470A) with a mix of random 6 mers and oligo (dT) primers. After mixing well generated cDNA templates with primers/probes and PerfectStart Green qPCR SuperMix (Transgen), RT-qPCR was performed with the Bio-Rad CFX Connect Real-Time PCR Detection System (Bio-Rad). The housekeeping gene, GAPDH, was used as a loading control. Primers were listed in Table 2 (**Supporting information**).

### Western blotting

All the exosomes and cells were lysed by RIPA bufer with the inhibitors of protease and phosphatase at 4 °C for 30 min, and then centrifuged at 12,000 g for 30 min at 4 °C. Te mass of the sample loading was adjusted to the same according to their protein concentrations that were detected by BCA Protein Assay Kit. The samples were separated by SDS-PAGE and transferred to polyvinylidene difuoride membrane after boiled for 5 min. The membranes block by 5% not-fat milk at room temperature for 1 h and incubated with related primary antibodies at 4 °C overnight. With several wash by Tris-bufered saline with 0.05% Tween-20, secondary antibodies incubated with the membranes at room temperature for 1 h. NcmECL Ultra (P10100, NCM Biotech) was applied for chemiluminescent exposure of the blot.

### Lentivirus construction and MSCs transfection

The peptide sequences for Lamp2b-CXCR4 expression are listed in the supporting information. Lentivirus containing a purinomycin resistance gene, cas9 protein gene, luciferase gene, sgRNA for USP5/USP8 (sgRNA sequence for USP5 is 5’-GTGTACTATACGGGAAACAGCGG-3’, sgRNA sequence for USP8 is 5’-TTTCAACCGAAACTGCTACCAGG) or gene for USP13, USP42 or USP 51 was constructed by Shanghai Jikai Biotechnology Co., LTD. The virus titer used to transfect MSCs was 1 × 10^7^.

### Cell proliferation detection

Cells were stained using a dye such as CFSE at a working concentration and then co-incubated with the different designated treatment for 24 h, after which the proliferation of the cells was observed using flow cytometry, where cells with attenuated CFSE fluorescence intensity were considered to be proliferating cells.

### Bioinformatics analysis

The mRNA expression data of USP5 in different cancer types, different stage and corresponding AML patients were obtained from the online analysis tool of Timer 2.0 database (http://timer.cistrome.org/) and the online analysis tool of the UALCAN database (https://ualcan.path.uab.edu/index.html). The surival data of AML patients were obtained from the online analysis tools of GEPIA 2 database (http://gepia2.cancer-pku.cn/#survival).

### Pre-processing of scRNA-seq data

The scRNA-seq data for AML samples (accession number GSE235923) were obtained from the GEO database (http://www.ncbi.nlm.nih.gov/geo). The raw output data were processed with the “Seurat” R package (version 4.3.0; http://satijalab.org/seurat/) for each individual sample. In each sample, low-quality cells that did not meet the following criteria were excluded: (1) 250 ≤ nCounts; (2) 200 ≤ nFeatures; (3) percentage of mitochondrial genes ≤ 20. Also, to avoid the effect of doubled cells, we excluded from the data doubled cells identified using “scDblFinder” (version 1.12.0; https://plger.github.io/scDblFinder/) R package.

### Data integration and the dimensionality reduction

All samples were merged with the “AddSamples” function into one Seurat object. The merged Seurat object was normalized and scaled by regressing out UMI count and percentage of mitochondrial genes. For dimensionality reduction, the top 3000 highly variable genes (HVGs) were determined using the “FindVariableGenes” function. Dimensionality reduction was then performed using PCA and UMAP plots were generated by the “RunUMAP” function with the first 100 PCs as input, determined by visualizing the drop off in PC variance explained using the “ElbowPlot” function in Seurat. The batch effects were removed by the “Harmony” R package (version 1.2.0; https://github.com/immunogenomics/harmony) based on the top 50 PCA components identified.

### Cell-clustering and annotation

The clustering analysis was performed based on the integrated joint embedding produced by Harmony with the Louvain algorithm after computing a shared nearest-neighbor graph with the Louvain algorithm that was implanted in the “FindClusters” function of the Seurat package. The identified clusters were visualized on the 2D map produced with the UMAP method. To annotate the cell clusters, DEGs with high discrimination abilities between the groups were identified with the “FindAllMarkers” function in Seurat using the default non-parametric Wilcoxon rank sum test with Bonferroni correction. The cell groups were annotated based on the DEGs and the well-known cellular markers from the literature. We then annotated clusters based on the average expression of genes in the following major cell types: MSCs (CD34), myeloid cells (CD68), T cells (PTPRC and CD3D), B cells (MS4A1), and fibroblasts (PDGFRB), endothelial cells (EPECAM1), and plasma cells (JCHAIN).

### RNA sequencing (RNA-seq) and bioinformatic analysis

Total RNAs were isolated from 1 × 10^6^ either the Mut-MSCs or Mut-MSCs with knock down USP5 gene by using TRIzol Reagent following the manufacturer’s instructions (Invitrogen). RNA libraries were were prepared and sequenced by the Illumina NovaSeq 6000. For analysis of RNA-seq results, RNA-seq reads quality was evaluated using FastQC (v0.11.9, https://www.bioinformatics.babraham.ac.uk/projects/fastqc/) and aligned to the mouse genome GRCm38 by HISAT2 (v2.2.1, https://daehwankimlab.github.io/hisat2/). FeatureCounts (v2.0.1, http://subread.sourceforge.net/) was used to quantitate the transcriptome using the GTF annotation files. Differential analyses were performed to the count files using the R packages DESeq2 (v1.28.1, https://bioconductor.org/packages/release/bioc/html/DESeq2.html), following standard normalization procedures. The differentially expressed genes (DEGs) were identified with adjusted *p* value < 0.05 and absolute log2 fold-change > 1 and plotted with R packages ggplot2 (v3.3.2, https://cran.r-project.org/web/packages.

/ggplot2/index.html). Heatmaps were generated using pheatmap package (v1.0.12, https://cran.r-project.org/web/packages/pheatmap/index.html) in R (v4.0.2, https://cran.r-project.org). Gene-set enrichment analysis was performed using the GSEA software (v4.1.0, https://www.gsea-msigdb.org/gsea/index.jsp).

### Preparation of exosomes and other engineered Rexo

In 10 cm cell culture dishes, 6 × 10^6^ MSCs were planted. Exosomes were isolated using differential ultracentrifugation with a Beckman Coulter Allegra X-15R (rotor SX4750) at 1,000 × g for 10 min to remove cells, followed by 14,000 × g for 30 min to eliminate debris, and finally pelleted at 100,000 × g for 70 min (Beckman Optima XE-90, Type 70 Ti rotor). Washed exosomes were resuspended in sterile PBS for downstream applications. To prepare Exosome-CP, the exosomes were infused with CP05-PSN peptide in a 2:5 ratio of mass for overnight, Overnight incubation allows CP05-mediated anchoring to exosomal CD63 via high-affinity binding, as previously described [29]. Exosomes-CP were gained from the supernatant via 100,000 g for further 60 min at 4 °C and washed with sterile 1×PBS for 2 times. At last, The Exosomes-CP were resuspended with 1×PBS for subsequent experiments.

### Quantification of exosomes and other engineered Rexo

The protein concentrations of exosomes and other engineered exosomes were measured. After washing, indicated engineered exosomes were lysed with radioimmunoprecipitation assay (RIPA) buffer at 4 °C for 30 min and then centrifuged for 30 min at 12,000 g at 4 °C. The supernatant containing the total protein was transferred to a new centrifuge tube. Protein was quantified using the BCA Protein Assay Kit (Thermo Fisher Scientific) in accordance with the manufacturer’s protocol.

### Exosomes encapsulated with USP5-IN-1 via electroporation

USP5-IN-1 was dissolved in ddH_2_O with 0.1% DMSO to aid solubility, and the drug concentration was 10 mg mL^− 1^. The drug was mixed with exosomes in a 1:1 ratio of mass in 400 mM sucrose solution. By an electroporation system (Gene Pulser X cell, Bio Rad, USA), 400 µL mixture was electroporated in 0.2 cm cuvettes via exponential pulse (voltage: 500 V; capacitance: 250 µF; pulse duration: 10 ms). The loading efficiency (LE) was 12.5 ± 2.1%, and drug content (DC) was 0.12 mg USP5-IN-1 per mg exosome protein, determined by HPLC.

### Characterization of exosome size and transmission electron microscopy (TEM)

One milliliter of 30 ng mL^− 1^ exosomes were taken for the measurement of the particle size and polydispersity index by Malvern laser particle size analyzer (Zetasizer Nano.

ZSP). For further identifcation of the sizes and morphology of exosomes were washed by ddH_2_O, deposited on copper mesh and then observed by TEM (HT7700-SS/FEI Tecnai G20 TWIN).

### Analysis of USP5-IN-1 loaded in USP5@Exosome-CP in vitro by HPLC

Ultrasound of USP5@Exosome-CP was performed in methanol solution following centrifugation (11 0000 g, 30 min). Then, the supernatants were filtered (0.2 μm filters) for HPLC (LC-2030 C Plus, designed by Shimadzu Corporation in Japan). A C18 (250 × 4.6 mm, 5 μm particle size) HPLC packed column was used as the chromatographic column. The mobile phase was CH3OH 0.5%TFA/H_2_O 0.5% TFA (1:1, *V/V*), the flow rate was 1.0 mL min^− 1^, and the detection wavelength was 254 nm.

### Immunofluorescence and confocal microscopy

Slice or cells were fixed in 4% paraformaldehyde for 15 min and then kept in a blocking buffer for 1 h. Then, the cells were incubated with primary antibodies overnight at 4 °C, followed by staining with secondary antibodies. Nuclei were stained with DAPI. After that, the cells were visualized using an LSM710 laser scanning confocal microscope.

### Mice

C57BL/6J female mice were obtained from Hunan Slyke Jingda Laboratory Animal Co. Ltd. in Hunan, China, and *JAK2*^V617F^ genetic mutation mice were obtained from Yuan Zhou laboratory. These mice were bred and maintained in a specific pathogen-free (SPF) barrier facility. All animal studies were conducted under the approval of the Hubei Provincial Animal Care and Use Committee and followed the experimental guidelines established by the Animal Experimentation Ethics Committee of Huazhong University of Science and Technology. This study was approved by the Ethics Committee of Huazhong agriculture University (HZAUMO-2024-0334).

### Construction of a mouse model of myeloproliferative neoplasms

*JAK2*^V617F^ genetic mutation mice BM c-kit^+^ cells were obtained: mice were killed by cervical dislocation, femur, tibia and ilium were isolated, BM cells were collected, and c-kit + cells were sorted out by incubation with CD117 magnetic beads. Construction of primary mouse mutant cells: luciferase-labeled gene vector was constructed using retroviral system, and the retroviral vector was packaged in HEK293T cells, and then the viral supernatant was obtained and infected with the BM c-kit + cells of mice. To construct a mouse model for MPN transplantation, primary c-kit + cells containing the luciferase gene were collected from infected mice and implanted into homozygous female recipients irradiated with a lethal dose of γ-rays (8.0 Gy) via a tail vein. The MPN transplanted mice were evaluated for the changes in peripheral blood counts by tail vein blood sampling at regular intervals after transplantation, and the size of the spleen and the degree of myelofibrosis in the mice were observed.

### Bioluminescence and fluorescence imaging

After anesthetizing C57BL/6J female mice (6 ~ 8 weeks old) with 1% pentobarbital sodium, they were intraperitoneally injected with firefly luciferin (150 mg kg − 1; Sigma-Aldrich; CAS: 103404-75-7). After 15 min, mice were imaged using the Bruker In Vivo MS FX PRO Imager with 3 min exposure times for acquiring luminescent or fluorescent images.

### Histopathological assays

The organs from mice were collected, immediately fixed in 10% (*m/V*) neutral-buffered formalin, and embedded in paraffin. Sections approximately 5 μm thick were cut and mounted on slides, and were examined using H&E staining and visualized under a light microscope.

### Data statement

Sample sizes were predetermined based on previous experience, using at least three groups of mice. All experiments were replicated at least twice to confirm findings. Statistical analyses were conducted using a two-tailed unpaired *t*-test or one-way ANOVA as appropriate. Mice were randomly assigned to treatment groups, and, where possible, treatment groups were blinded until statistical analysis. No animals or potential outliers were excluded from the data sets presented in this study. Data used and analyzed during the study are available from the corresponding author on reasonable request.

## Electronic supplementary material

Below is the link to the electronic supplementary material.

Supplementary Material 1

## Data Availability

No datasets were generated or analysed during the current study.
