# Supplementary material for: USP5 inhibition via bone marrow-targeted engineered exosomes for myeloproliferative neoplasms therapy
Source: J Nanobiotechnology. 2025 Jul 11;23:501. doi: 10.1186/s12951-025-03588-4 (PMC12247442; doi:10.1186/s12951-025-03588-4)
Supplement: Supplementary file 1 — Supplementary Material 1 [file 12951_2025_3588_MOESM1_ESM.docx]

**Supporting information**

**USP5 Inhibition via Bone Marrow-Targeted Engineered Exosomes for Myeloproliferative Neoplasms Therapy**

Wenjun Wang^1,2,3^, Yufeng Jiang^4^, Donglei Zhang^1^, Xian Zhang^1^, Qian Liang^2,3,5^, Jun Shi^2,3*^, Yuan Zhou^2,3*^, Fuling Zhou^1*^.

^1^Department of Hematology, Zhongnan Hospital of Wuhan University, Wuhan University, Wuhan, 430071, China

^2^State Key Laboratory of Experimental Hematology, National Clinical Research Center for Blood Diseases, Haihe Laboratory of Cell Ecosystem, Institute of Hematology & Blood Diseases Hospital, Chinese Academy of Medical Sciences & Peking Union Medical College, Tianjin 300020, China

^3^Tianjin Institutes of Health Science, Tianjin 301600, China

^4^School of Information and Mathematics, Yangtze University, Jingzhou, Hubei 434000, China

^5^Zhoukou Central Hospital, Zhoukou, China

* Correspondence: zhoufuling@whu.edu.cn (F. Zhou), yuanzhou@ihcams.ac.cn (Y. Zhou), or shijun@ihcams.ac.cn (J. Shi);


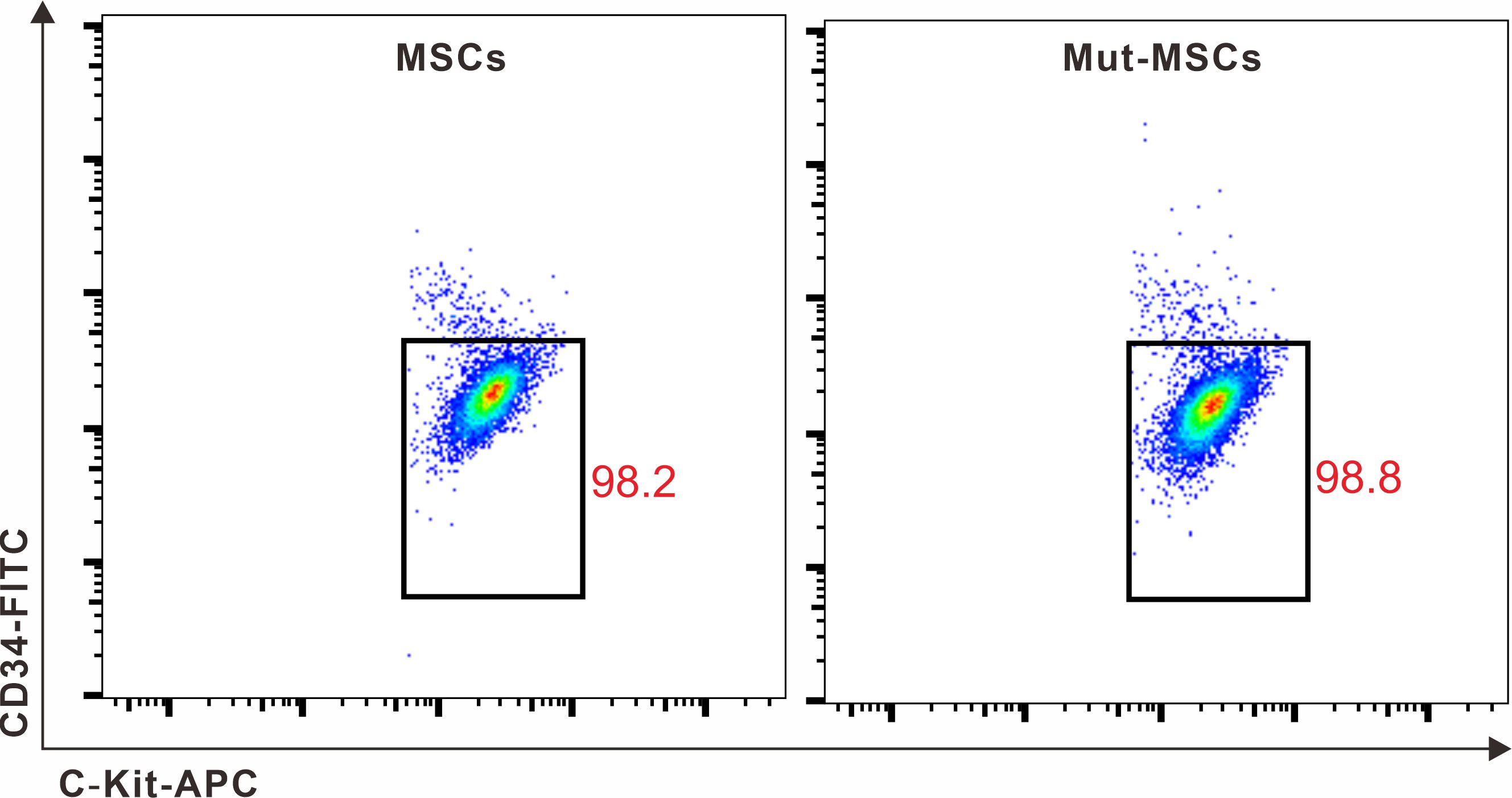


Figure S1. Flow cytometry characterization of the purity of MSCs isolated from normal mice and mice harboring the JAK2^V617F^ mutation.


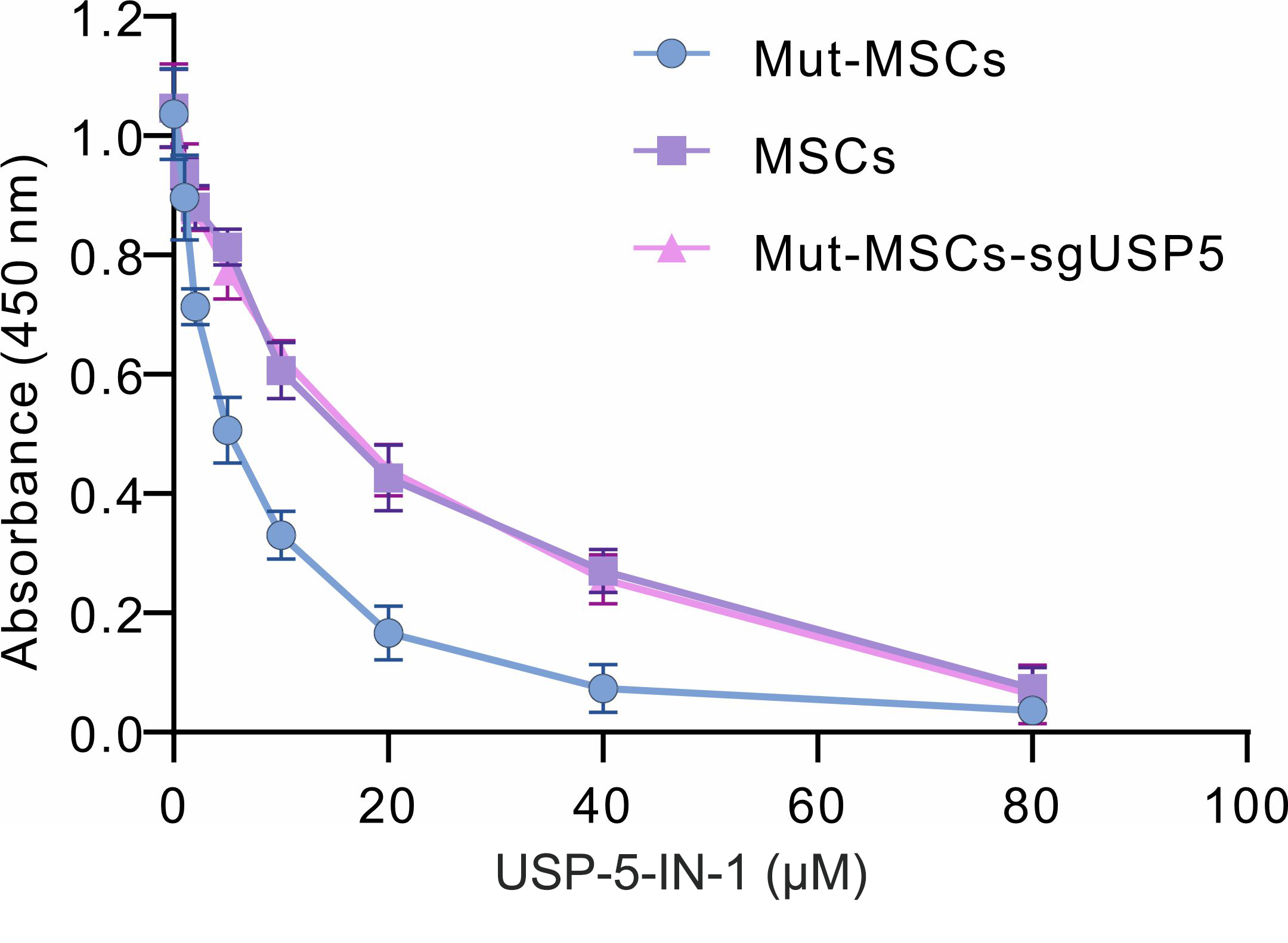


Figure S2. Effect of USP5 inhibitor on death induction in different MSCs.


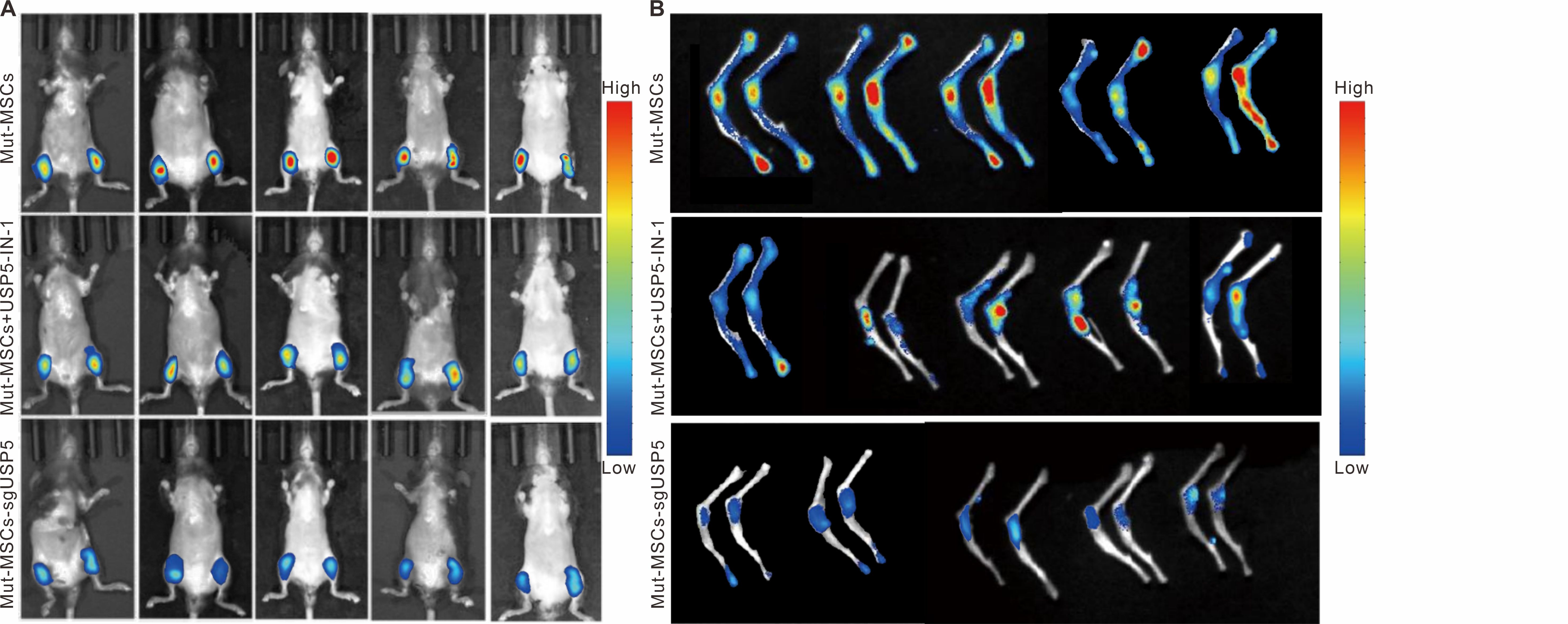


Figure S3. Effect of MPN model formation in different designated treatment groups. (A) MPN models were constructed using luciferase-expressing Mut-MSCs cells, and the effects of MPN modeling in different designated treatment groups were observed by *in vivo* imaging of small animals. (B) Bioluminescence imaging of isolated bone marrow from the indicated treatment groups (not whole-body imaging).


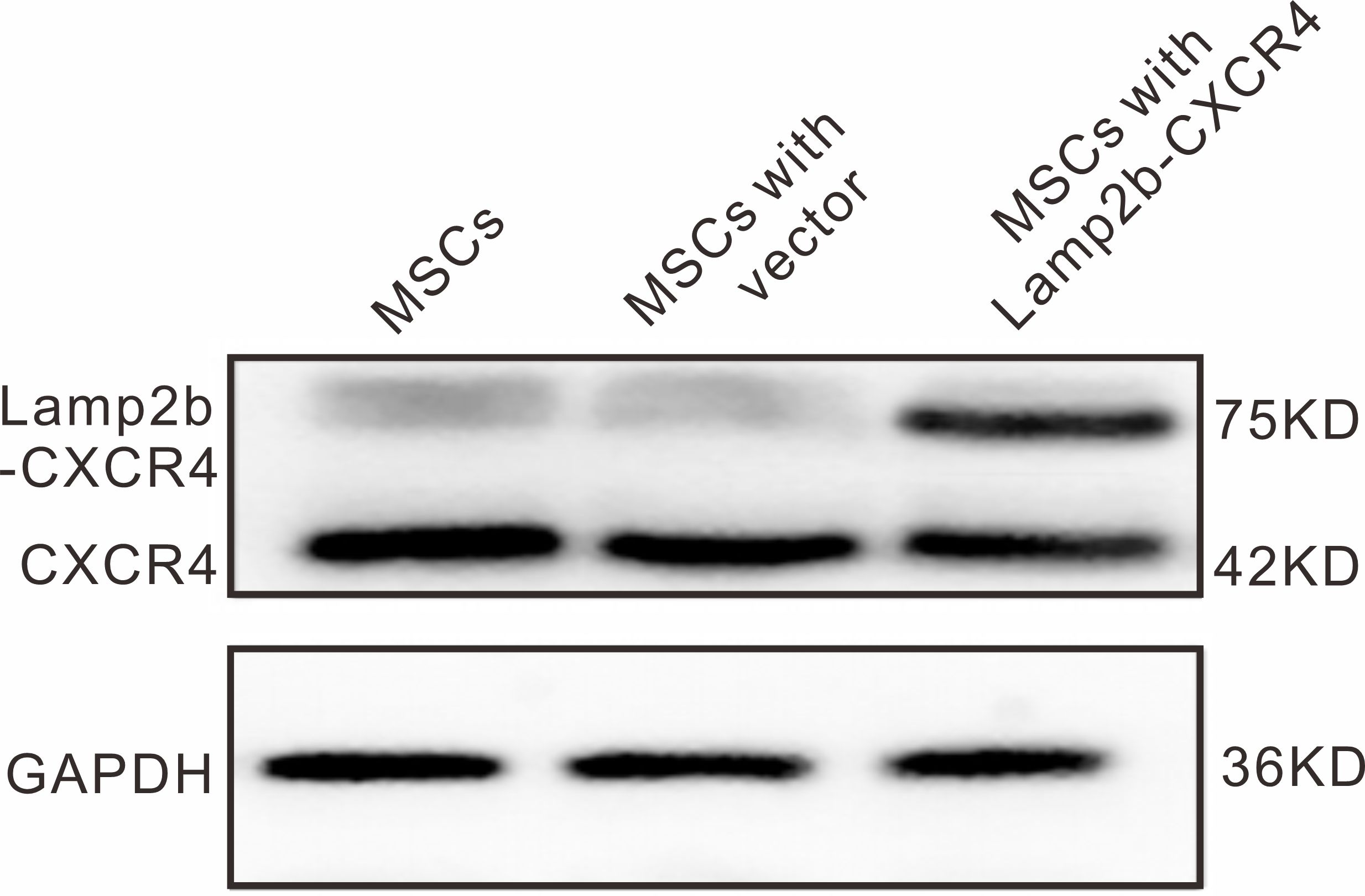


Figure S4. Western blotting to verify MSCs overexpressing the Lamp2b-CXCR4 fusion protein.


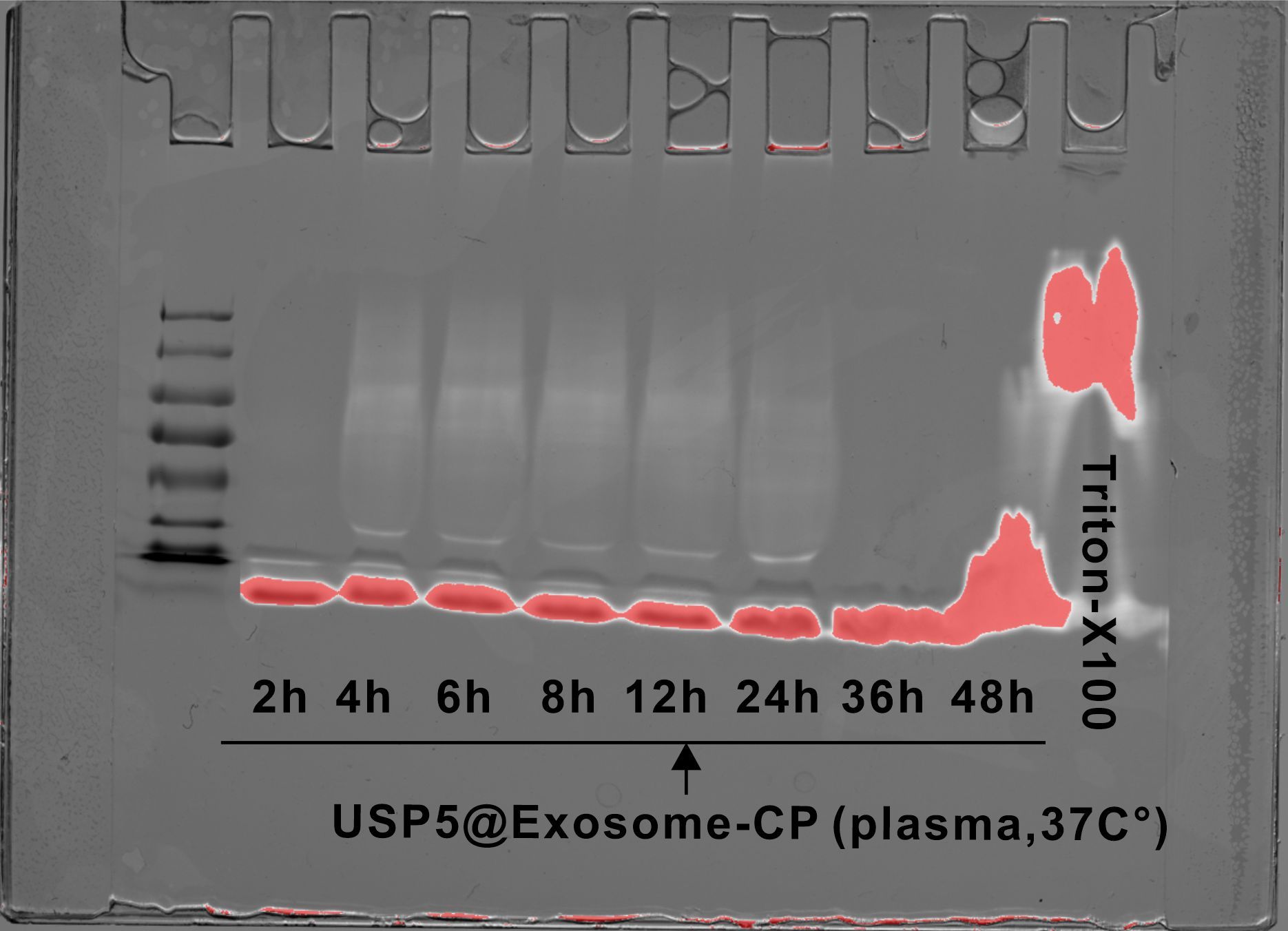


Figure S5. Naive SDS-PAGE to detect stability of USP5@Exosome-CP in plasma at 37 ℃ for different time points. TritonX-100 destroys the nanostructure of USP5@Exosome-CP and release free FITC labeled CP peptide, serving as a positive control in the experiment.


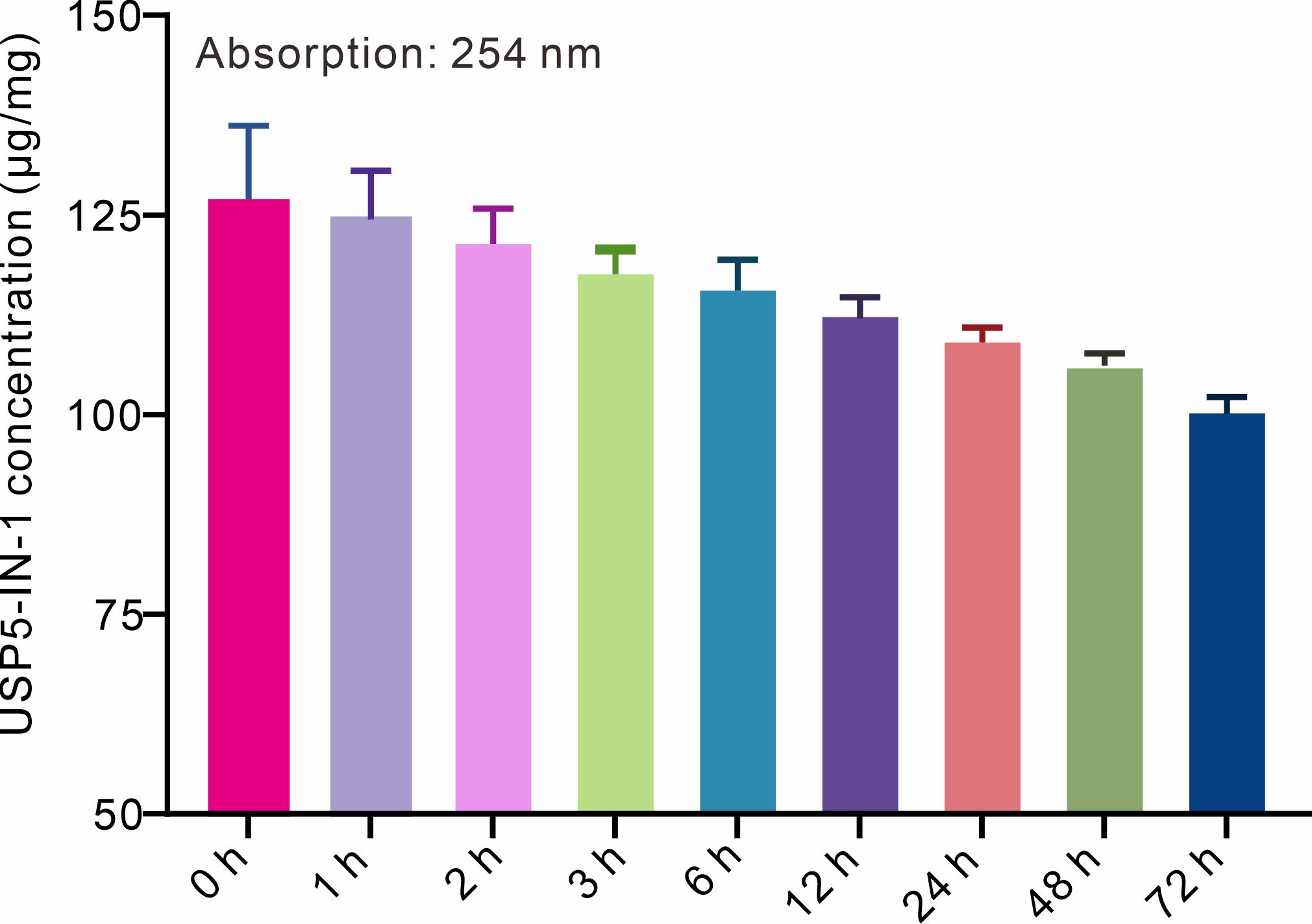


Figure S6. HPLC identification of USP5-IN-1 remaining after incubation of USP5@Exosome-CP with 10% mouse serum for different time.


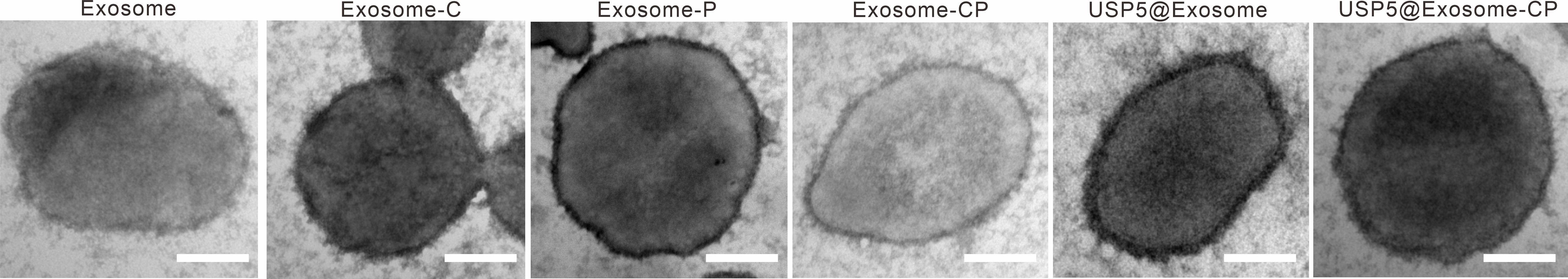


Figure S7.Transmission electron microscopic observation of the morphological characteristics of different groups of engineered exosomes. Scale bar is 50 nm.


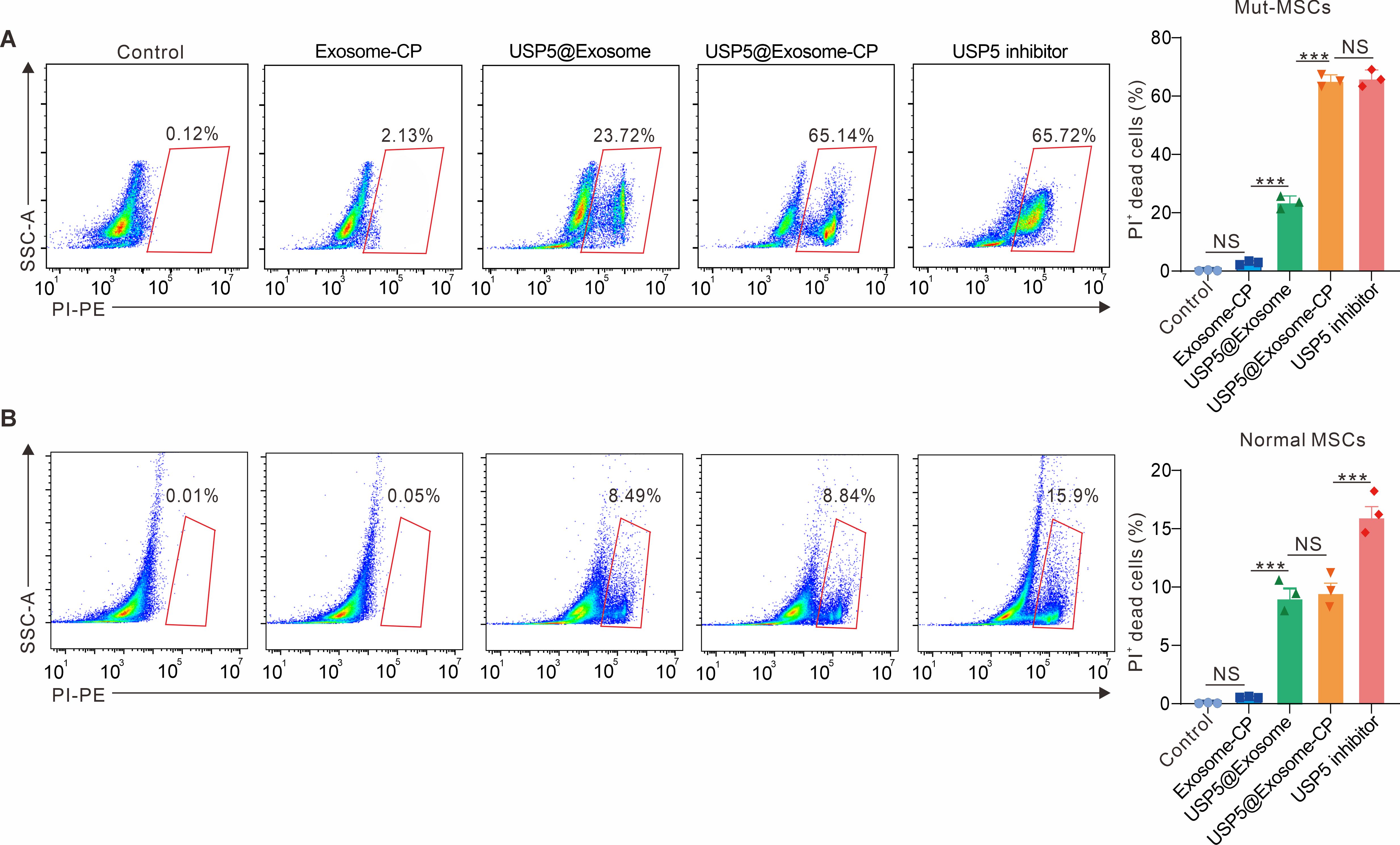
Figure S8. Flow cytometry statistics on the killing properties of Mut-MSCs and normal MSCs in MPN model by different designated treatment groups. (A) Statistics of the proportion of PI^+^ cells in Mut-MSCs after different treatment group. (B) Statistics of the proportion of PI^+^ cells in normal MSCs after different treatment group.

Table1. the fusion protein sequence of Lamp2b-CXCR4 used in the study.

| Protein name | Peptide sequence |
| --- | --- |
| Lamp2b-CXCR4 | MEPISVSIYTSDNYSEEVGSGDYDSNKEPCFRDENVHFNRIFLPTIYFIIFLTGIVGNGLVILVMGYQKKLRSMTDKYRLHLSVADLLFVITLPFWAVDAMADWYFGKFLCKAVHIIYTVNLYSSVLILAFISLDRYLAIVHATNSQRPRKLLAEKAVYVGVWIPALLLTIPDFIFADVSQGDISQGDDRYICDRLYPDSLWMVVFQFQHIMVGLILPGIVILSCYCIIISKLSHSKGHQKRKALKTTVILILAFFACWLPYYVGISIDSFILLGVIKQGCDFESIVHKWISITEALAFFHCCLNPILYAFLGAKFKSSAQHALNSMSRGSSLKILSKGKRGGHSSVSTESESSSFHSSGGGGSMCLSPVKGAKLILIFLFLGAVQSNALIVNLTDSKGTCLYAEWEMNFTITYETTNQTNKTITIAVPDKATHDGSSCGDDRNSAKIMIQFGFAVSWAVNFTKEASHYSIHDIVLSYNTSDSTVFPGAVAKGVHTVKNPENFKVPLDVIFKCNSVLTYNLTPVVQKYWGIHLQAFVQNGTVSKNEQVCEEDQTPTTVAPIIHTTAPSTTTTLTPTSTPTPTPTPTPTVGNYSIRNGNTTCLLATMGLQLNITEEKVPFIFNINPATTNFTGSCQPQSAQLRLNNSQIKYLDFIFAVKNEKRFYLKEVNVYMYLANGSAFNISNKNLSFWDAPLGSSYMCNKEQVLSVSRAFQINTFNLKVQPFNVTKGQYSTAEECAADSDLNFLIPVAVGVALGFLIIAVFISYMIGRRKSRTGYQSV |

Table 2. The prime sequence of deubiquitinating enzyme family used in the study.

| Gene | Forward primer | Reverse primer |
| --- | --- | --- |
| UCHL1 | TCTGTCTGAAACGGAGAAGCTGTC | CATGGTTCACTGGAAAGGGCATTC |
| UCHL3 | AGACCAGTATGCGCAGTGTT | TCCAATCGTTCCACAGGCAT |
| UCHL5 | ACAACTTGCAGAGGAACCCAT | CTGGTGCTCAGCTAAGGTCT |
| BAP1 | GAGAGGACGAGGAGTGGACAGATA | CTGCCATCAGGTTGAAGCGAATG |
| JOSD1 | AGACAAGGCCAAATCTGAATCCTT | ATGCTCTTCTTGTGGGGTGTT |
| JOSD2 | GAAGCGAGACCAAGATCACCC | GGGCTAGCCTCTTGCAGATT |
| ATAXIN3 | GTGCTCAGCATTGCCTGAATAACC | TTCTCAGCCTCTCCTCTTCATCCA |
| OTUD1 | CGATTCCACATCATCCCCGA | CCCTCAATGAGTGGGCTGAA |
| OTUD3 | CTTCGTGGAAGATGACATTCCC | AAAGGGGCATTAAGCTGATGG |
| OTUD4 | AGGCAGACATAGACTTGGCTTCAG | TGGTCCTCTTAGGCTCAGGTTCA |
| OTUD5 | AGGACGGTGCCTGTCTATTTC | CATCAGATAGTCCATGCAATGCT |
| OTUD6A | GCCAACTCACCCACCATTGTCA | CCGAATTGTAGTGCTCTCCGAAGT |
| OTUD6B | GCAGCTCACGGAAGATGTTG | AATCCGAGGTGGTTGATTCTCA |
| OTUD7A | ACAGCAGAACAAGGAGGAGGAATG | AGTATGTGGGCGAGGACAAAGAC |
| OTUD7B | CGGAAGATGAGTGGCAGAAGGAA | TGAGCAAGGACGAAGACATGGAAT |
| OTUDB1 | GCTGTGCAGAATCCTCTGGT | AAGCCAAACGCTCGGTAGAA |
| OTUDB2 | AACTCAGCAAAAGATTCACCTCG | TCATTTGGGGTCTGTAGCACA |
| YOD1 | GCGATACTGGGAAAGACAAACG | AGCGGATCGTAGTGAATGCC |
| OTULIN | ACGATGACAAGGAGAAGGGAAAGG | CGGACAGCATAGGCAAGAAGGAA |
| TRABID | TGTGTGGGTGTTGTGGAAGG | GCGTATGGCCAGATGTACCA |
| VCPIP1 | AACCAGAGCACGTCAACACA | CATGCACTAGGCAGTGTCCA |
| PSMD7 | GGACGCCAACGTCAAAAACT | AGTCCCCACTGTAGTGTCCT |
| PSMD14 | AAACATGGTCGTGCTGGAGT | CCAGTTCCTGACTGTGGCAT |
| AMSH | CCCTGAAACCTGGAGCGTTA | AGGTTTCAATGCCTTTGGCG |
| PRPF8 | TGGCCGGAGTGTTTCCTTAC | CATATCGCTTGGCCTGCAAC |
| BRCC3 | GCAGGCGGTTCATCTTGAGT | GACTGTGCGCATTTCCGTTC |
| EIF3H | CACACGGAGGATGATGCTGACTT | TGAGGACGACAGACTCTTCAATGG |
| EIF3F | CACCAACTGCTTTTCGGTGC | TGTCATGGCCTGTGGCATAC |
| MYSM1 | TTCTAATAGCAGCGTCCCCA | ACTCCTCAGCGATGAAGCAA |
| COPS6 | GCCTATGCCCTATGTCACTGTCTT | CCAATGCCTTGTCGGTCGTAGA |
| MPND | AAGAAGATGTGCTGGCTGGG | TTGTCCATACGCTTCCCTGG |
| USP1 | TAATGGGCTTTCAAGAGGCAGT | TCTTCTCGCAGCTTACTGGG |
| USP2 | GTGCTCCACCTGAAGCGATTCT | GGACTTCGGCAGTAGGCTGTATAG |
| USP3 | GTTTGCTCCTTAAGAGATTGCC | TCTCAGTGGGAATTGAACGTAT |
| USP4 | CCAAAAGGTGAAAGGCCAGC | TGCTCAGGTGCTTCTCACAG |
| USP5 | GAAGACGGACAAGACGATGACTGA | ACCTGGACCACGGAGTTGAGATA |
| USP7 | TCGTCGCACATTGAGACGG | CTTGTCGGCATGGTTGGGAAT |
| USP8 | GCCTGCTGTAGCTTCAGTTC | AAGATCTTCTGCGCGCTGTG |
| USP9X | GACCGGGACACCCAAAAGAT | CATGGGGACTTCGCTGACTT |
| USP9Y | AGTTTGCTGTGAAGCCCTGGAA | AGAACTGCTCCTGTGCCAACTG |
| USP10 | CTGAAGCCGTTGAAAAAGATGAG | TCAGCCTCTGCGTTAGAGTTG |
| USP11 | GCAGAACCATAAACGACGAAAT | CACAGATCTGAGATATTGCCCT |
| USP12 | CAGTCTCCAAATTCGCCTCCA | GTGCTCGTTGACCGGAAACT |
| USP13 | CAGTCTGCCCTTGGCATTTAC | CCTCTTCTCTTAGGGAGCACAG |
| USP14 | ACCTCCAATGGTGTTCAAAGC | CATCCTTCAGGGTTCCTCCTT |
| USP15 | TCAGCCATTCAGTGTTTGAGC | TTTCACCTCTCATTCCTAAGGGA |
| USP16 | TGGCTCCTTTTTGTACCCTTAA | GTACCACTGTGTTCAACAACTC |
| USP18 | CAGGAGTCCCTGATTTGCGTG | CAGAGGCTTTGCGTCCTTATC |
| USP19 | TGGAGATGCTAGGAGAGTGTC | CGCAGCTTAACAATCACCTCAT |
| USP20 | TGGACTGCATAGGGGAGGTG | GGCAAGCCCATAGGTTAGGTC |
| USP21 | AACTCCATGTTACGACCTTTGC | AAGGGGACCTCTAGGACGAGA |
| USP22 | TCTTTCTGTCGGATAGGCACC | GCCCTGAGTAAAACTCCTGGA |
| USP24 | GCTGGAAAGCCGCGTTTTG | CAAGTCTGGCTAAGTAGGTGGA |
| USP25 | TCTCGAAACCCCTATGACAGAA | TGAATAACTGCACTAAACCAGCA |
| USP27X | GACATTGAGCAAATTGCCAAAGA | AAGTCCCGGCACTGAACAC |
| USP28 | GGGTCCGAGAAGGAAAGCC | CACGGAACGATCCGAAGGAAG |
| USP29 | GCTGGGTGATAATGTTACAGGC | TGAGTAAGGATGTGTCGTCTCT |
| USP30 | CCTCACCCCACGACCAATC | GAGAAAGGCTGTCAAAGGTGT |
| USP31 | TTCTTTGCCAATTCCTCTACCC | CACAGCCACACCGATCCTC |
| USP32 | GTCCCAGATACACTCAGGAAGT | AGCGAGAGAAGGTAAAAGCATC |
| USP33 | GGACCACAGCACCATACACTC | GCTGCAAGCATAACACCATACT |
| USP34 | CTGGTTGCCTATGAAGGCTTG | AGCTTGATGCAGTTTTTCGACA |
| USP36 | CCAACAGCGGCAATGCTATC | CATCGCATCAATGGTGTACCG |
| USP37 | AGTCAGCCTGCTCGTTCACTA | AAGTCAACATTAGGCGGCTTT |
| USP38 | GCCCCTCAAGCGGATGATT | GGGTCGTCAGGTCAAACATGG |
| USP39 | GTCACTGCCCGTACTTGGATA | GTATGCGTTGATGTGCGAGAG |
| USP40 | TGACCGACTGGTTAAAGCAGC | GCTAGTATCCTTGTAGCGTTCAC |
| USP42 | AGGCGGTCTCACCTGAAGA | CACTGGCCCTAATGGAAGTGT |
| USP45 | ATGCGGGTAAAAGATCCATCAAA | ACGTTAGACCTACAGCAATGTCA |
| USP46 | ATGACTGTCCGAAACATCGCC | TTGACCAATCCGAAGTAGTGTTC |
| USP47 | GATGTGATTCCCTTGGATTGCT | AACCCCATTGGTGTATCTTCTTC |
| USP48 | CAGAGGAAACCCGAATTGCTT | GTGGCTCCCAGGTTAGTCAAG |
| USP49 | AGTTCCGGGAATGTTTCCTGA | CTCCTTACTGACAACTCTGCG |
| USP26 | ACCAATGAGGACCACAACCA | CACTCTGGTTCACACCCCTT |
| USP35 | GGAGTGGGTTCTAAGGCAAGAA | GTAGGACGAAGTCACCACGG |
| USP43 | GCAGGCCCGAGGTTCATC | TGTGAGGTGCCAGCTGTACT |
| USP44 | CCGTGCCTGGTTACTGACAT | CTGCTTCTGGGCTTCTGTGA |
| USP50 | CCTCTGCAGCGTATCTCCAC | TCATCAGGTAGGCAAAGGCG |
| USP51 | TGCGGATGATCTATCAGCGTTTCA | CACAGGACAGACAAGAGTGGAGTC |
| USP52 | GGCTTCCTCTTCCACATGCT | AGAAACTGCCTCCAGCACC |
| USP53 | CTGTTACACCAGCCGACACT | AAGGGGCTGTAAGTGGCATC |
| USP54 | GCAGAATCAGGGGAGGAGTG | TTACACAGAGCCAGAGCTGC |
| CYLD | ACCCTACTGGGAAGAACGGAT | CGGTCTTGGATGTACTGTCCTAT |
| USP17le | GGTCTTTGGAGACATGGTGGT | TCTGGGGCATCAGGAGATGA |
| USPL1 | CAGGGACTGATGTAGGGATATCTT | GTGACTCAGAGCCCAAAGGAT |
| DESI2 | AGATTCCTCGCTGGATCAACCG | GGCTGCATCCTCTGCTTCTTCT |
